# *VHL*-*HIF-2α* axis-induced *SEMA6A* upregulation stabilized β-catenin to drive clear cell renal cell carcinoma progression

**DOI:** 10.1038/s41419-023-05588-4

**Published:** 2023-02-04

**Authors:** Jing Ji, Yuxin Xu, Mengru Xie, Xingbei He, Dexu Ren, Teng Qiu, Wenwen Liu, Zefeng Chen, Wen Shi, Zhen Zhang, Xiujun Wang, Weiling Wang, Jinming Ma, Qilan Qian, Aixin Jing, Xinhui Ma, Jingting Qin, Yuanyuan Ding, Ting Geng, Jiayan Yang, Zhichao Sun, Wei Liu, Shunfang Liu, Bin Liu

**Affiliations:** 1 Jiangsu Key Laboratory of Marine Pharmaceutical Compound Screening, College of Pharmacy, Jiangsu Ocean University, Lianyungang, 222005 China; 2 Department of Pathology, The Second People’s Hospital of Lianyungang, 41 Hailian East Road, Haizhou, Lianyungang, 222006 Jiangsu PR China; 3 Department of Oncology, Tongji Hospital of Tongji Medical College, Huazhong University of Science and Technology, Jiefang Road 1095, Wuhan, 430030 PR China

**Keywords:** Cancer prevention, Cancer therapy

## Abstract

*SEMA6A* is a multifunctional transmembrane semaphorin protein that participates in various cellular processes, including axon guidance, cell migration, and cancer progression. However, the role of *SEMA6A* in clear cell renal cell carcinoma (ccRCC) is unclear. Based on high-throughput sequencing data, here we report that *SEMA6A* is a novel target gene of the *VHL*-*HIF*-*2α* axis and overexpressed in ccRCC. Chromatin immunoprecipitation and reporter assays revealed that *HIF-2α* directly activated *SEMA6A* transcription in hypoxic ccRCC cells. *Wnt*/β-catenin pathway activation is correlated with the expression of *SEMA6A* in ccRCC; the latter physically interacted with *SEC62* and promoted ccRCC progression through *SEC62*-dependent β-catenin stabilization and activation. Depletion of *SEMA6A* impaired *HIF-2α*-induced *Wnt*/β-catenin pathway activation and led to defective ccRCC cell proliferation both in vitro and in vivo. *SEMA6A* overexpression promoted the malignant phenotypes of ccRCC, which was reversed by *SEC62* depletion. Collectively, this study revealed a potential role for *VHL*-*HIF-2α*-*SEMA6A*-*SEC62* axis in the activation of *Wnt*/β-catenin pathway. Thus, *SEMA6A* may act as a potential therapeutic target, especially in *VHL*-deficient ccRCC.

## Introduction

Renal cell carcinoma (RCC) is the second leading cause of death in patients with urinary cancer worldwide, with a frequency of 1/30,000, accounting for 2–3% of all adult cancers [1], and one of the tenth most common cancers globally [2]. The most common subtype of RCC is clear cell renal cell carcinoma (ccRCC) [3], which accounts for about 75% of all RCC cases [4]. It is characterized by malignant epithelial cells with clear cytoplasm, dense nest-like growth, and complicated dendritic blood vessels [5]. Currently, surgery is the main strategy for the treatment of ccRCC [6, 7]. However, the cancer is not sensitive to postoperative radiotherapy and chemotherapy, accompanied by high metastasis and recurrence rates [8]. In addition, clinically effective methods for early prevention, early diagnosis, post-diagnosis treatment, and prognostic evaluation of ccRCC are yet lacking [9]. Therefore, clarifying the molecular mechanisms of ccRCC will provide clues for the early diagnosis and targeted therapy of ccRCC.

Interestingly, ccRCC is triggered by the biallelic inactivation of the tumor suppressor gene Von Hippel-Lindau (*VHL*) in renal epithelial cells, which deregulates the hypoxia-inducible factors (*HIFs*), including *HIF-1a* and *HIF-2α* [10, 11]. *VHL* can form a classical E3 ubiquitin ligase complex with elonginB, elonginC, Cullin2, and *Rbx1* [12]. This complex can target *HIF-2α* for degradation through the ubiquitin-proteasome pathway [13]. Under normoxia, the proline residues of *HIF-2α* are hydroxylated by prolyl hydroxylase, and the hydroxylated *HIF-2α* is then recognized by this E3 ubiquitin ligase complex, followed by sequential degradation [14]. Conversely, *VHL* failed to recognize *HIF-2α* under hypoxia, resulting in its accumulation [15]. The accumulated *HIF-2α* is associated with the constitutively expressed *HIF-1β* to form a heterodimer, which is then transferred to the nucleus. Subsequently, the expression of the downstream genes, vascular endothelial growth factor A (*VEGFA*) and cyclin D1 (*CCND1*) [16], is activated, and several physiological processes, such as angiogenesis, cell metabolism, cell proliferation, and cell apoptosis are regulated [17–19]. *VHL* mutations are observed in ~70–80% of ccRCC [20], and the Cullin2-*VHL* E3 ubiquitin ligase complex cannot be formed, resulting in the stabilization of the *HIF-2α* protein irrespective of the cellular oxygen supply [21]. The stabilized *HIF-2α*, together with *HIF-1β*, causes the abnormal activation of the downstream oncogenes and promotes the occurrence and development of ccRCC [22].

Herein, the transcriptional profiling from *HIF-2α* silenced, and *VHL* overexpressed ccRCC cells was integrated, which facilitated, which in turn led to the identification of *SEMA6A* as a direct downstream target gene of the *VHL*-*HIF-2α* axis. Next, we revealed that *SEMA6A* is required for the integrity of the *HIF-2α*-β-catenin signaling pathway in ccRCC, and the genetic deletion of *SEMA6A* resulted in impaired proliferation ability in ccRCC cells by repressing *SEC62*-dependent β-catenin stabilization and activation.

## Materials and methods

### Data collection

GSE149005, GSE32297, GSE68417, and GSE79683 were downloaded from the gene expression omnibus (GEO) database on the NCBI website. (The R software limma package was used to perform differential expression analysis, and *p* < 0.05 and |logFC| > 1.2 were set as the cutoff criteria. Venn diagrams were drawn to obtain intersecting genes, and Gene Ontology (GO) and Kyoto Encyclopedia of Genomes (KEGG) pathway analyses were performed. The mutation frequencies and copy number alterations in the *SEMA6A* gene were observed by genomic analysis of renal clear cell carcinoma (The Cancer Genome Atlas Firehose Legacy, 538 samples) through the cBioPortal online tool (http://www.cbioportal.org/). The transcript levels of *SEMA6A* were obtained from the GEPIA database (http://gepia.cancer-pku.cn) between normal kidney tissue and KIRC. The expression of *SEMA6A* in different subtypes of kidney renal clear cell carcinoma (KIRC) was analyzed via the UALCAN online tool (http://ualcan.path.uab.edu/).

### Cell culture and tissue samples

All cell lines used in this study were purchased from American Type Culture Collection (ATCC) and cultured in an appropriate medium containing 10% fetal bovine serum (FBS, Gibco, Grand Island, NY, USA) and supplemented with penicillin (100 U/ml) and streptomycin (100 µg/ml), in a 37 °C incubator with 5% CO_2_. All cell lines were negative for mycoplasma during the experiment. 6 paired fresh human ccRCC samples and adjacent normal kidney tissues were obtained from the Second People’s Hospital of Lianyungang. The study was approved by the Medical Ethical Committee of the Second People’s Hospital of Lianyungang, and informed consent was obtained from all subjects or their relatives.

### Cell proliferation, colony formation, and migration assays

For cell counting kit-8 (CCK-8) analysis, 5 × 10^4^ cells were seeded in a 96-well plate and CCK-8 reagent was added to the wells; the absorbance was measured at 450 or 490 nm. For bromodeoxyuridine (BrdU) assay, the cells were seeded in a 96-well plate and incubated for 24 h. Then, the cell culture medium was discarded, the cell fixative solution was added to each well, and the cells were fixed, denatured, and incubated with anti-BrdU and TMB substrate. The absorbance was measured at 450 nm on a microplate reader. For colony formation assay, about 1000 cells were seeded in each well of a 12-well plate, and the seeded 12-well plate was cultured for about 2 weeks in a 5% CO_2_ and 37 °C incubator. Cells were washed with phosphate-buffered saline (PBS), fixed with paraformaldehyde for 15–30 min, and stained with 0.05% crystal violet or Giemsa to count the number of colonies. To assess the cell migration ability, 5 × 10^5^ cells were seeded in 12-well plates for 24 h. When the cell density was about 80%, a 10-μl pipette tip was used to draw a line perpendicular to the bottom of each well, washed three times with PBS to remove the floating cells. Images were taken under the microscope at the same position at 0, 24, and 48 h after scratching, and the migration area was calculated using ImageJ software.

### Structure modeling and protein docking

Full-length sequences of *SEMA6A* and *SEC62* were uploaded onto the I-TASSER webserver, and the modeling was run with default settings. Five structure clusters were obtained as results for each protein, the final models were selected according to the overall conformations, and the C-scores were calculated by the server. The protein-protein docking was performed using the Cluspro online server. Ligplot software was used to analyze the binding force between the two proteins, and Pymol software was used for three-dimensional (3D) protein-binding conformation imaging.

### Immunoprecipitation (IP) and glutathione-S-transferase (GST)-pull-down

The IP protocol has been described previously [23]. Briefly, cells were lysed with IP buffer (100 mM NaCl, 20 mM Tris-HCl pH 8.0, 0.5 mM EDTA, 0.5% (v/v) Nonidet P-40) with protease inhibitor cocktail and phosphatase inhibitor for 30 min on ice. The cells were sonicated, and the lysate was collected by centrifugation at 14,000 rpm for 10 min. For endogenous IP, the filtered supernatant was incubated with either anti-SEMA6A or anti-SEC62 or IgG and protein A/G beads at 4 °C overnight on a rotating wheel. The immunoprecipitates were washed three times with IP buffer and denatured at 95 °C for 5 min. The final sample was separated by 12% SDS-PAGE and detected by immunoblotting. The interaction between *SEMA6A* and *SEC62* was assessed by GST-pull-down assay. Briefly, GST or GST-SEC62 was expressed in BL21 cells and purified using glutathione-Sepharose beads. Equivalent amounts of GST or GST fusion proteins were resuspended in reaction buffer (20 mM HEPES, pH 7.5; 5 mM MgCl_2_, 0.5 mM EDTA, 0.05% NP40, 1 mM DTT and 130 mM KCl,) containing 0.2 mg/ml bovine serum albumin (BSA). Then, 1 mg FLAG-*SEMA6A*-transfected cell lysate was added to each mixture, followed by rotation at room temperature for 1 h. Subsequently, the beads were boiled in a sample buffer to elute the bound proteins. The final sample was separated by SDS-PAGE and detected by immunoblotting.

### Western blot

Cells were harvested and lysed with SDS-containing lysis buffer (100 mM Tris-HCl, pH 6.8, 100 mM DTT, 1% SDS, 10% glycerol). An equivalent amount of protein from each sample was separated by SDS-PAGE and transferred to polyvinylidene fluoride (PVDF) membranes. After blocking with 5% nonfat milk for 1 h, the membranes were incubated with the indicated primary antibodies at 4 °C overnight. After washing with Tris buffered saline Tween (TBST) buffer (50 mM Tris-HCl, 0.15 M NaCl and 0.05% Tween 20), the membrane was incubated with the secondary antibody at 25 °C for 1 h. Then, the immunoreactive bands were developed using enhanced chemiluminescence reagent with β-Actin as a loading control. The primary antibodies were as follows: anti-*HIF-2α* (sc-8712, Santa Cruz), anti-*SEMA6A* (sc-398302, Santa Cruz), anti-*SEC62*(ab244335, abcam), and anti-β-Actin(sc-8432, Santa Cruz).

### RNA inhibition and quantitative real-time polymerase chain reaction (qRT-PCR)

Lentiviral human shRNA plasmids targeting *SEMA6A* or *HIF-2α* were purchased from Sigma. The lentiviral particles were used to infect ccRCC cells and screened by puromycin for ~2 weeks. RNA was extracted from the samples using the RNeasy extraction kit and reverse transcribed into cDNA using the reverse transcription kit according to the manufacturer’s instructions. RT-PCR was performed using Light Cycler 480, and *β-actin* was used as an internal reference gene. The relative expression of the target gene was analyzed using the 2^−ΔΔCt^ method.

### Luciferase activity and chromatin immunoprecipitation (ChIP) assays

The TOP/FOP flash reporters were purchased from Beyotime. The promoter region of human *SEMA6A* gene was amplified from the genomic DNA of A498 cells and cloned into the pGL4.15 vector (Promega, Madison, WI, USA). After transfection, the cells were harvested and lysed, and the luciferase activity was assayed using the Dual Luciferase Assay System (Promega). The luciferase activity was normalized to Renilla luciferase activity. For ChIP assay, cells were fixed with formaldehyde, and DNA was sheared to fragments at 100–500 bp by sonication. Then, the supernatant was incubated with antibodies against *HIF-2α* or normal serum IgG at 4 °C overnight.

### Xenograft assays

For subcutaneous xenografts, 3 × 10^6^ cells in 300 μl PBS were injected subcutaneously into the flanks of 6-week-old male nude mice. Tumors were measured every 7 days after injection, the tumor volume was calculated according to the formula (length × width^2^)/2, and the mice were sacrificed 4 weeks after inoculation. All animal experiments were approved by the Institutional Animal Ethics Committee of Jiangsu Ocean University, and animal care was in accordance with the institutional guidelines.

### Statistical analyses

All experiments were repeated at least three times. Data were expressed as mean ± standard deviation (SD). The statistical analysis was performed using GraphPad Prism 9.0 software (USA, San Diego, CA). The differences between the groups were calculated using either the Student’s *t* test or one-way analysis of variance using Tukey’s test. Statistical significance was expressed as *p < 0.05, **p < 0.01, and ***p < 0.001, respectively.

## Results

### Identification of the potential target genes of the *VHL*-*HIF-2α* axis in ccRCC cells

**Fig. 1:**
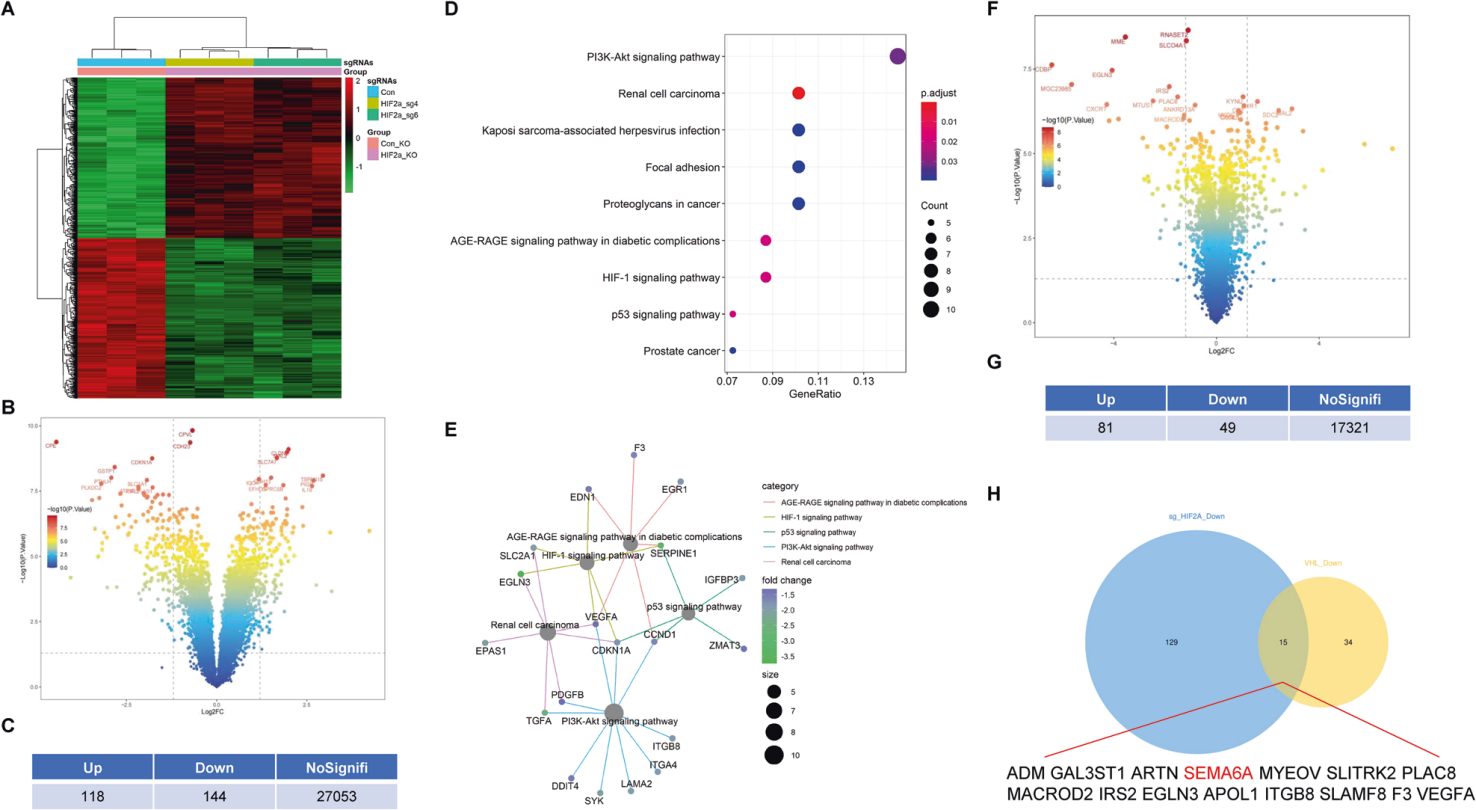
Identification of the potential target genes of the *VHL*-*HIF-2α* axis in ccRCC cells. **A** Heatmap of the DEGs expression regulated by *HIF-2α* in ccRCC cells in GSE149005. *p* < 0.05, | log2FC| > 1.2. **B** Volcano map of DEGs expression regulated by *HIF-2α* in GSE149005. **C** Number of up- and downregulated DEGs in GSE149005. **D** KEGG pathway analysis of the downregulated genes in GSE149005 through the clusterProfiler R package. **E** Gene network diagram generated by the clusterProfiler R package showed the key genes associated with the enrichment pathway. **F** Volcano map of the DEGs in GSE32297. *p* < 0.05, |log2FC| > 1.2. **G** Number of up- and downregulated DEGs in GSE32297. **H** Venn diagram shows the intersection between the downregulated genes in GSE149005 and GSE32297 databases.

In order to explore new substrate genes of *HIF-2α* in ccRCC, we first analyzed the transcriptional profiling of *HIF-2α*-regulated genes in ccRCC cells using a previously published RNA sequencing (RNA-seq) dataset (GSE149005). GSE149005 contains a transcriptional gene expression profile of 786-O cells treated with sgRNAs to *HIF-2α* or scrambled control. *p* < 0.05 and |logFC| > 1.2 were set as the cutoff criteria. A total of 118 genes were upregulated, and 144 genes were downregulated after *HIF-2α* was depleted (Supplementary Tables 1 and 2). The differentially expressed genes (DEGs) were exhibited by the heatmap and volcano map, respectively (Fig. 1A–C). The KEGG analysis showed that these downregulated genes were significantly enriched in the *PI3K*-*AKT* signaling pathway, RCC, *HIF-1* signaling pathway, and p53 signaling pathway (Fig. 1D). The gene-concept network generated by the R package clusterProfiler showed that *VEGFA*, *CCND1*, and *CDKN1A* were the key genes linked to these enriched pathways (Fig. 1E). Thus, these results highlighted the critical roles of *HIF-2α* and its target genes in the regulation of these pathways. As the tumor suppressor *VHL* protein promotes the ubiquitination and degradation of *HIF-2α*, we speculated that forced expression of *VHL* in ccRCC cells might indirectly inhibit the expression of *HIF-2α* target genes. Interestingly, the overexpression of *VHL* inhibited the expression of *CXCR4*, a downstream gene of *HIF-2α*. In order to further determine the genes regulated by *VHL*, we used a previously published GEO dataset to analyze the transcription profile of *VHL*-regulated genes in ccRCC cells. Using the same cutoff criteria, we found that 81 genes were upregulated and 49 genes were downregulated in the presence of exogenous *VHL* protein (Fig. 1F, G and Supplementary Tables 3 and 4). Next, we identified that the genes were downregulated after knocking out *HIF-2α* and *VHL* overexpression. As depicted in Fig. 1H, 15 genes were identified using these screening criteria. Together, our screening strategy showed that these genes might contain new target genes of the VHL-HIF-2α axis in ccRCC cells.

### *SEMA6A* is a direct target gene of *HIF-2α*

**Fig. 2:**
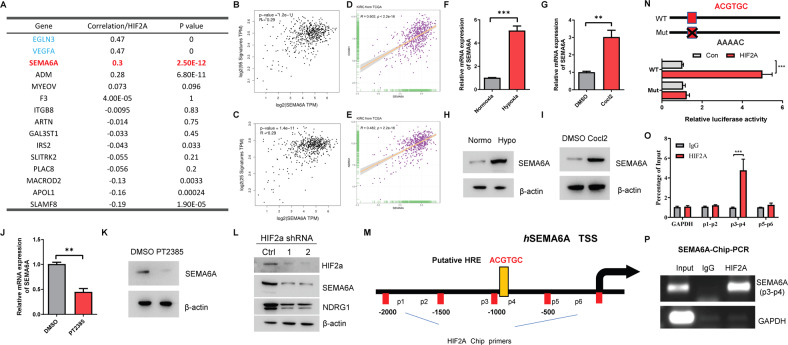
*SEMA6A* is a transcriptional target of *HIF-2α* in ccRCC. **A** mRNA correlation between the mRNA level of *HIF-2α* and its 15 putative target genes in TCGA KIRC dataset was analyzed using the GEPIA platform (http://gepia2.cancer-pku.cn/). **B** The mRNA correlation between *SEMA6A* and 95 hypoxia metagenes in the TCGA KIRC database. **C** The mRNA correlation between *SEMA6A* and 25 hypoxia metagenes in the TCGA KIRC database. **D** mRNA correlation between *CCND1* and *SEMA6A* in the TCGA KIRC database. **E** mRNA correlation between *NDRG1* and *SEMA6A* in the TCGA KIRC database. **F**, **G** Relative mRNA and protein levels of *SEMA6A* in A498 cells under either normoxia or hypoxia conditions. ***p < 0.001. **H**, **I** Relative mRNA and protein levels of *SEMA6A* in A498 cells in response to CoCl_2_ treatment. **p < 0.01. **J**, **K** Relative mRNA and protein levels of *SEMA6A* in A498 cells in response to PT2385 treatment. **p < 0.01. **L** Protein levels of *HIF-2α*, *SEMA6A*, and *NDRG1* in A498 cells transfected with control shRNA or shRNAs targeting *HIF-2α*. **M** Schematic representation of the human *SEMA6A* gene promoter and the putative *HIF-2α* binding site. TSS transcription start site. **N** Human *SENA6A* promoter contains a potential *HIF-2α* binding site, highlighted in red. Mutant sites are marked with a cross. FLAG-*HIF-2α* was co-transfected with the indicated plasmids into 293T cells for 36 h. Luciferase activity was then measured. ***p < 0.001. **O** ChIP-qPCR assay showed the enrichment of *HIF-2α* in the putative *HIF-2α* binding site of the *SEMA6A* promoter region. ***p < 0.001. **P** ChIP-PCR assay showed the occupation of *HIF-2α* in the putative *HIF-2α* binding site of the *SEMA6A* promoter region.

The correlation between the mRNA expression of *HIF-2α* and these 15 potential target genes in ccRCC was identified from the TCGA database (Fig. 2A), including *EGLN3*, *VEGFA*, *SEMA6A*, and *ADM*. Among these, *VEGFA* is a well-known *HIF-2α* target gene and *EGLN3* positively regulates the mRNA expression of *HIF-2α* [24]. Therefore, we focused on *SEMA6A*, a protein known as the regulator of axonal guidance [25], because it might be another target gene of *HIF-2α* in ccRCC. We first compared the correlation of SEMA6A expression with two previously reported sets of hypoxia metagenes [26, 27]. We found that the expression of SEMA6A showed a positive correlation with these two groups of hypoxic metagenes in the TCGA-KIRC database, suggesting that SEMA6A may also have some expression characteristics of hypoxia metagenes (Fig. 2B, C and Supplementary Tables 5 and 6). Interestingly, the mRNA expression level of *SEMA6A* was also significantly correlated with both *CCND1* and *NDRG1* (Fig. 2D, E), two known *HIF-2α* target genes, suggesting that *SEMA6A* is associated with the *HIF-2α* signaling pathway in ccRCC. Moreover, both hypoxia and hypoxia mimics significantly induce the expression of *SEMA6A* in both A498 and 786-O cells (Fig. 2F–I and Supplementary Fig. 1A–D). Then, we utilized a *HIF-2α* antagonist PT2385 to treat A498 cells and found that PT2385 treatment reduced both the mRNA and protein levels of *SEMA6A* (Fig. 2J, K). Silencing the expression of *HIF-2α* by two different shRNAs consistently decreased the levels of *NDRG1* and *SEMA6A* proteins (Fig. 2L). These data suggested that *SEMA6A* might be a putative *HIF-2α* downstream target gene in ccRCC. Notably, a classic hypoxia response element (HRE) was detected in the promoter region of the human *SEMA6A* gene (Fig. 2M), prompting us to test whether this site is essential for *HIF-2α* recognition and *SEMA6A* expression. In a luciferase assay, we found that *HIF-2α* significantly promotes the luciferase activity of the *SEMA6A* wild-type (WT) promoter (Fig. 2N). Moreover, ChIP analysis showed that *HIF-2α* binds to this putative HRE site in the *SEMA6A* promoter (Fig. 2O, P). These data indicated that *SEMA6A* is a direct target gene of *HIF-2α* in ccRCC.

### *SEMA6A* is overexpressed in ccRCC patients

**Fig. 3:**
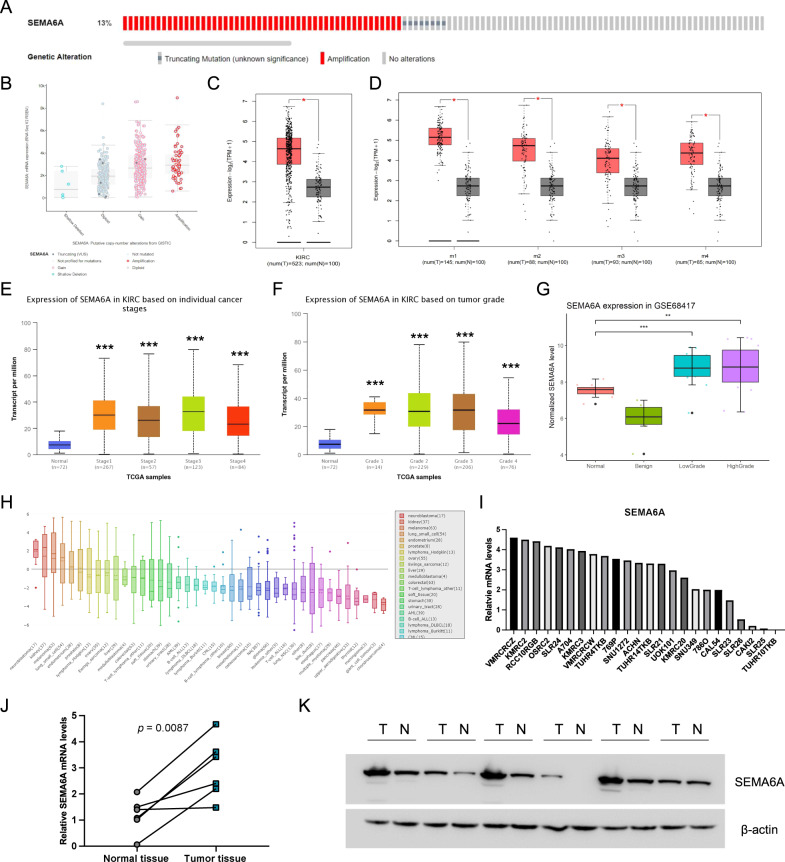
*SEMA6A* is overexpressed in ccRCC patients. **A** Mutation of *SEMA6A* in ccRCC was obtained through the online cBioPortal database (http://www.cbioportal.org/). **B** Copy number and mutation types of the expression of *SEMA6A* in ccRCC. (TCGA, Firehose Legacy; 538 total samples; Genome map selection: Mutations, Putative copy-number alterations from GISTIC, mRNA expression *z*-scores relative to diploid samples (RNA-Seq V2 RSEM). **C** mRNA expression of *SEMA6A* in the TCGA database obtained through the GEPIA website (http://gepia.cancer-pku.cn). Red represents ccRCC tissue and black represents normal tissue. *p < 0.05. **D** Correlation between *SEMA6A* expression and ccRCC subtypes from TCGA database obtained through the GEPIA website (http://gepia.cancer-pku.cn). Red represents ccRCC tissue and black represents normal tissue. *p < 0.05. **E** Correlation between *SEMA6A* expression and the tumor stage of ccRCC patients was obtained through the UALCAN database (http://ualcan.path.uab.edu). **F** Correlation between *SEMA6A* expression and the tumor grade of ccRCC patients was obtained through the UALCAN database (http://ualcan.path.uab.edu). **G** Box plot of the difference in the expression level of *SEMA6A* in the GSE68417. Red represents normal tissues, green represents benign tissues, blue represents low-grade ccRCC tissues, and purple represents high-grade ccRCC tissues. **p < 0.01, ***p < 0.001. **H** mRNA expression of *SEMA6A* in cell lines from different cancer types was obtained through the CCLE website (https://sites.broadinstitute.org/ccle). **I** Bar graph of *SEMA6A* mRNA expression in several kidney cancer cell lines. **J**, **K** The mRNA and protein levels of *SEMA6A* in six paired ccRCC tissues and adjacent normal kidney tissues.

Next, we used the online cBioPortal database to investigate the expression and mutation of *SEMA6A* in ccRCC and found that the gene was mutated in 58/446 (13%) samples. The main genetic changes included gene amplification (50 cases, 11.2%) and mutations (8 cases, 1.79%) (Fig. 3A). The copy number and mutation types are shown in Fig. 3B. We also explored the mRNA expression of *SEMA6A* in ccRCC from TCGA using the online GEPIA database. Compared to 100 normal renal tissues, the mRNA expression of *SEMA6A* in 523 ccRCC tissues was significantly increased (Fig. 3C). Also, the mRNA expression of *SEMA6A* in different subtypes of ccRCC was significantly higher than that in the normal renal tissues (Fig. 3D). Moreover, searching the online UALCAN database revealed that the mRNA expression level of *SEMA6A* in different pathological stages and tumor grades of ccRCC was higher than that in normal renal tissues (Fig. 3E, F). Consistent with these observations, higher mRNA expression of *SEMA6A* in ccRCC than in normal and benign tissues was confirmed in an independent cohort of ccRCC patients (GSE68417) (Fig. 3G). We also found that *SEMA6A* is highly expressed in kidney cancer cell lines compared to most other cancer types exploring the Cancer Cell Line Encyclopedia (CCLE) database (Fig. 3H, I). We then collected 6 paired fresh human ccRCC samples and adjacent normal kidney tissues, and then performed qRT-PCR and western blot experiments. Our results showed that the mRNA and protein levels of SEMA6A were higher in most ccRCC samples when compared with adjacent normal tissues (Fig. 3J, K). Taken together, these data show that SEMA6A is overexpressed in ccRCC patients.

### Targeting *SEMA6A* inhibits ccRCC proliferation

**Fig. 4:**
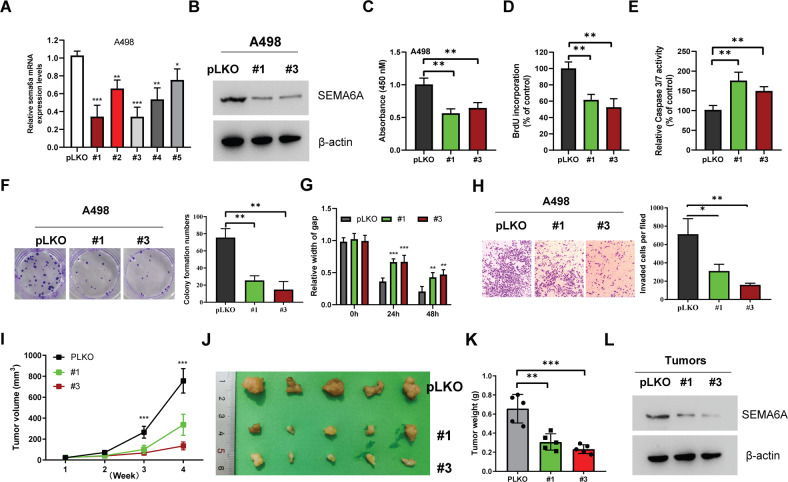
Depletion of *SEMA6A* inhibits ccRCC malignancy both in vitro and in vivo. **A** Relative expression levels of *SEMA6A* mRNA in A498 cells transfected with indicated shRNAs. *p < 0.05, **p < 0.01, ***p < 0.001. **B** Western blot analysis of *SEMA6A* protein in A498 cells stably expressing indicated shRNAs. **C** Proliferation ability of A498 cells from **B** was determined by CCK-8 assay. **p < 0.01. **D** Relative number of BrdU-incorporated cells in A498 cells from **B**. **p < 0.01. **E** Relative Caspase3/7 activity in A498 cells from **B** **p < 0.01. **F** Colonies of A498 cells from **B**. *p < 0.05, **p < 0.01. **G** Migration ability of A498 cells from **B**. **p < 0.01, ***p < 0.001. **H** Invasion ability of A498 cells from **B**. **p < 0.01, ***p < 0.001. **I**–**L** A498 cells stably expressing indicated shRNAs were injected subcutaneously into 6-week-old male nude mice (*n* = 5), and tumor growth curves were drawn weekly (**I**). After 4 weeks, mice were sacrificed by cervical dislocation. Then, the tumors were removed, photographed (**J**), weighed (**K**), and subjected to Western blot analysis to detect SEMA6A expression (**L**). **p < 0.01, ***p < 0.001.

To evaluate the biological function of *SEMA6A* in ccRCC, we suppressed *SEMA6A* expression in ccRCC cells. A498 cells were treated by lentiviral infections with five independent shRNAs against *SEMA6A*. Although all shRNAs could inhibit the expression of *SEMA6A*, #1 and #3 showed the best inhibitory effect (Fig. 4A). Silencing *SEMA6A* by #1 and #3 shRNAs also significantly decreased the level of *SEMA6A* protein (Fig. 4B). The CCK-8 assay showed that silencing *SEMA6A* can significantly inhibit the proliferation of A498 cell line (Fig. 4C). Consistently, *SEMA6A*-depleted cells show decreased numbers of BrdU-incorporating cells (Fig. 4D). Moreover, *SEMA6A* knockdown cells showed enhanced Caspase3/7 activity, suggesting increased apoptosis in these cells (Fig. 4E). Downregulation of *SEMA6A* also impaired the ability of A498 cells to form colonies (Fig. 4F). In addition, wound healing assays showed that *SEMA6A* knockdown significantly reduces the migratory and invasion abilities of A498 cells (Fig. 4G, H). A similar phenotype of decreased malignancy was observed in 786-O and Caki-1 cells with *SEMA6A* knockdown (Supplementary Figs. 2 and 3). To analyze the consequences of *SEMA6A* silencing in vivo, we subcutaneously engrafted A498 cells expressing control and *SEMA6A* shRNAs into nude mice and monitored tumor growth. Consistent with the in vitro results, *SEMA6A* depletion decreased the subcutaneous tumor volume, size, and weight (Fig. 4I–L). Taken together, these findings demonstrated that *SEMA6A* depletion inhibits ccRCC cell growth both in vitro and in vivo.

### *SEMA6A* activated the *Wnt*/β-catenin pathway via β-catenin stabilization

**Fig. 5:**
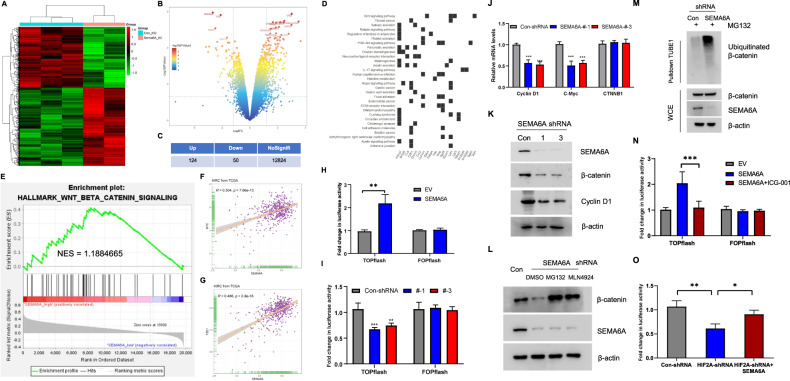
*SEMA6A* maintains the stability and activity of β-Catenin in ccRCC cells. **A** Heatmap showing DEGs expression regulated by *SEMA6A* in ccRCC cells. **B** Volcano plot showing differential genes regulated by *SEMA6A* in GSE149005. **C** Number of genes regulated by *SEMA6A* includes 124 up- and 50 downregulated genes. **D** Correlative signaling pathways enriched in genes downregulated by *SEMA6A*. **E** GSEA plot of *SEMA6A* enrichment in HALLMARK_WNT_BETA_CATENIN_SIGNALING signaling pathway. **F** mRNA correlation of *C-Myc* and *SEMA6A* in the TCGA KIRC database. **G** mRNA correlation of *FZD1* and *SEMA6A* in the TCGA KIRC database. **H** TOPFlash or FOPFlash were co-transfected with empty vector plasmid or *SEMA6A* into 293T cells, and the luciferase activity was measured after 36 h. **p < 0.01. **I** TOPFlash or FOPFlash were co-transfected with indicated shRNAs into 293T cells, and the luciferase activity was measured after 36 h. **p < 0.01, ***p < 0.001. **J** Relative mRNA levels of *Cyclin D1*, *C-Myc*, and *CTNNB1* in cells stably expressing the indicated shRNAs. ***p < 0.001. **K** Western blot analysis of lysates from cells in **J**. **L** Western blot analysis of A498 cells with or without *SEMA6A* depletion, followed by treatment with DMSO, MG132, and MLN4924 for 4 h, respectively. **M** A498 cells with or without *SEMA6A* depletion were treated with MG132 for 4 h. Ubiquitinated proteins were enriched using tandem ubiquitin-binding entity 1 (TUBE1) resin and subjected to immunoblot analysis using the indicated antibodies. **N** TOPFlash or FOPFlash was co-transfected with or without *SEMA6A* into A498 cells and then treated with or without ICG-001. The luciferase activity was measured after 36 h. ***p < 0.001. **O** TOPFlash or FOPFlash was co-transfected with indicated shRNAs into A498 cells, and then luciferase activity was measured after 36 h. *p < 0.05, **p < 0.01.

To further determine the mechanism of action of *SEMA6A* in ccRCC, we analyzed the transcriptional profiles of *SEMA6A*-regulated genes in ccRCC cells using the GSE79683 dataset. The dataset consists of the transcriptional gene expression profiles from *SEMA6A* WT and *SEMA6A* knockout (KO) mice. *p* < 0.05 and |logFC| > 1.2 were set as the cutoff criteria, and the DEGs were exhibited in both heatmap (Fig. 5A) and volcano map (Fig. 5B). Compared to WT control, 124 genes were upregulated, and 50 were downregulated in *SEMA6A* KO mice tissue (Fig. 5C and Supplementary Tables 7 and 8). The pathway enrichment analysis of these downregulated genes revealed that the *Wnt* signaling pathway is significantly suppressed in *SEMA6A* KO mice tissue (Fig. 5D). Then, we divided the sample data of KIRC in the TCGA database into high- and low-expression groups according to *SEMA6A* mRNA expression levels, and conducted GSEA. Compared to the low *SEMA6A* expression group, the *Wnt*/β-catenin signaling pathway was significantly enriched in the high *SEMA6A* expression (Fig. 5E). In agreement, the KIRC dataset from TCGA revealed that the mRNA level of *SEMA6A* was positively correlated with both *FZD1* and *Myc*, two well-known downstream target genes of the *Wnt*/β-catenin signaling pathway (Fig. 5F, G). These data prompted us to explore whether *SEMA6A* could regulate the *Wnt*/β-catenin signaling pathway. Thus, we conducted a TOPFlash/FOPFlash assay to assess whether *SEMA6A* affects the transcriptional activity of β-catenin/TCF complex. We also found that the ectopic expression of *SEMA6A* significantly increases TOPflash activity, but not FOPflash activity, compared to control vector expression (Fig. 5H). Conversely, silencing *SEMA6A* decreased the TOPflash activity without affecting the FOPflash activity (Fig. 5I). The decreased TOPflash activity could be attributed to the impaired transcriptional activity of the β-catenin/TCF complex, as observed by the declined mRNA expression of two β-catenin/TCF target genes: *CCND1* and *C-Myc* (Fig. 5J). Surprisingly, the mRNA level of *β-catenin* was unchanged in *SEMA6A*-depleted cells (Fig. 5J), while the immunoblotting results showed that inhibition of *SEMA6A* expression decreased the protein levels of both β-catenin and *CCND1* (Fig. 5K), suggesting that *SEMA6A* regulates the stability of β-catenin. In agreement, both the proteasome inhibitor MG132 and the pan-cullin inhibitor MLN4924 prevented the decline of β-catenin protein levels in *SEMA6A*-depleted cells (Fig. 5L), indicating that this decline was caused by enhanced proteolysis. Furthermore, depletion of *SEMA6A* increased the ubiquitination of β-catenin protein (Fig. 5M). Together, these results suggested that *SEMA6A* activates the *Wnt*/β-catenin signaling pathway by promoting the stability of β-catenin protein. Typically, the administration of ICG-001, a small molecule compound that selectively antagonizes β-catenin/TCF-mediated transcriptional activity, significantly reversed *SEMA6A*-induced TOPflash activation (Fig. 5N). In addition, ICG-001 administration also significantly reversed *SEMA6A*-induced A498 cell proliferation (Supplementary Fig. 6A), Caspase3/7 activation (Supplementary Fig. 6B) and colony formation in vitro (Supplementary Fig. 6C), suggesting *SEMA6A*-regualted β-catenin activation is related to ccRCC progression. Consistent with previous findings, *HIF-2α* depletion significantly reduced the transcriptional activity of β-catenin/TCF complex, which could be partially restored by *SEMA6A* overexpression (Fig. 5O), indicating that *SEMA6A* mediates *HIF-2α*-induced activation of the *Wnt*/β-catenin signaling pathway. In conclusion, the current results indicated that *SEMA6A* activates the *Wnt*/β-catenin signaling pathway in ccRCC.

### *SEMA6A* enhances the binding between *SEC62* and β-catenin

**Fig. 6:**
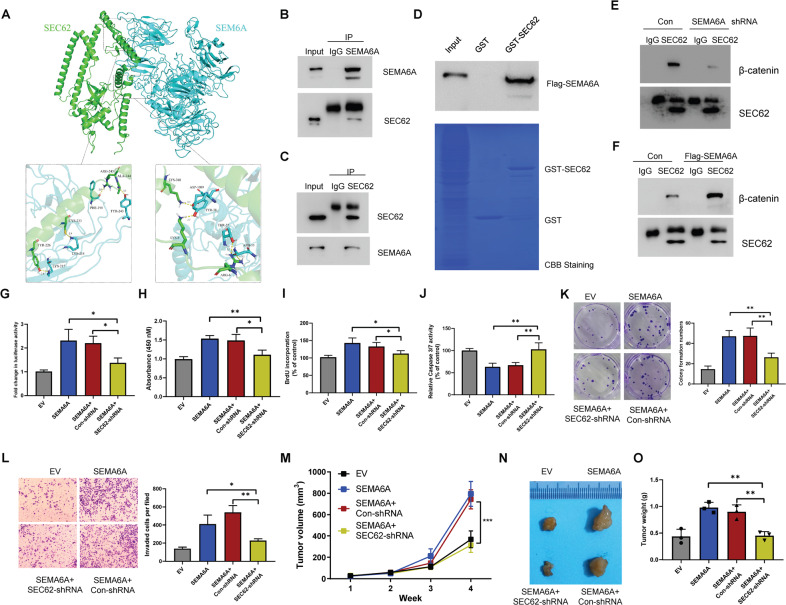
*SEMA6A* stabilizes β-catenin by enhancing *SEC62*-β-catenin interaction. **A** Structural model of the protein-protein interaction between *SEMA6A* (Cyan) and *SEC62* (Green). The yellow dotted lines represent hydrogen bonds. **B** Cell lysates from A498 cells were immunoprecipitated with IgG or anti-*SEMA6A* antibody and subjected to immunoblot analysis with indicated antibodies. **C** Cell lysates from A498 cells were immunoprecipitated with IgG or anti-*SEC62* antibody and subjected to immunoblot with indicated antibodies. **D** GST Sepharose beads coated with *E. coli* produced GST or GST-*SEC62* were incubated with purified FLAG-*SEMA6A*. The beads were washed, and the bound proteins were analyzed by Western blotting with indicated antibodies. **E** Cell lysates from A498 cells stable expressing con-shRNA or *SEMA6A*-shRNA were immunoprecipitated with SEC62 antibody and then subjected to immunoblotting with indicated antibodies. **F** Cell lysates from A498 cells stable expressing vector control or FLAG-*SEMA6A* were immunoprecipitated with *SEC62* antibody and subjected to immunoblotting with indicated antibodies. **G** A498 cells were co-transfected with TOPflash plasmid and the indicated shRNAs for 36 h. Then, the luciferase activity was measured. *p < 0.05. **H** Proliferation ability of A498 cells stable expressing the indicated shRNAs was determined by CCK-8 assay. **p < 0.01. **I** BrdU incorporation in A498 cells from **H** *p < 0.05. **J** Relative Caspase3/7 activity in A498 cells from **H** **p < 0.01. **K** Images of colony formation of A498 cells from **H**. **L** Migration ability of A498 cells from **H**. *p < 0.05. **M**–**O** A498 cells stably expressing indicated shRNAs were injected subcutaneously into 6-week-old male nude mice (*n* = 5), and tumor growth curves were drawn weekly (**M**). After 4 weeks, mice were sacrificed by cervical dislocation. The tumors were removed, photographed (**N**), and weighed (**O**). ***p < 0.001.

**Fig. 7:**
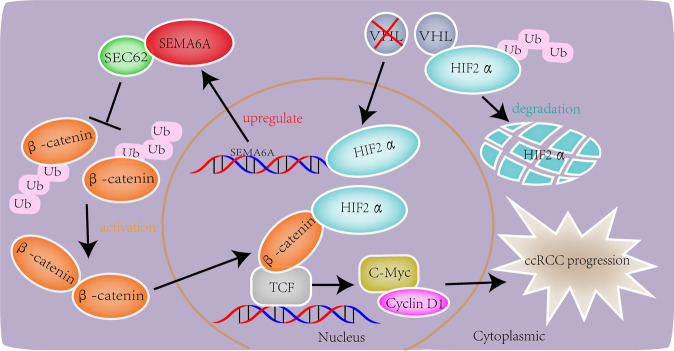
Working model. In this working model, we showed that hypoxia-dependent *HIF-2α* protein stabilization and activation-induced *SEMA6A* expression in ccRCC, which in turn bound to *SEC62* protein to inhibit the ubiquitination and degradation of β-catenin, thereby promoting the expression of multiple tumor-promoting genes and tumor progression.

To further investigate how *SEMA6A* regulates the stability of β-catenin protein, we searched for *SEMA6A*-interacting protein. A total of 82 putative *SEMA6A*-interacting proteins were identified using the BioGRID dataset (Supplementary Fig. 4), among which *SEC62* formed a protein complex with β-catenin and inhibited the degradation of β-catenin protein [28]. To evaluate whether *SEC62* is a binding partner of *SEMA6A*, we carried out structure-based computational analyses to show the interaction between *SEMA6A* and *SEC62*. Since the crystal structures for these two proteins are currently not available, we used the I-TASSER webserver for structural modeling. The protein-protein docking was performed using the Cluspro online server, a rigid protein docking algorithm based on a fast Fourier transform. Ligplot software was used to analyze the binding force between the two proteins, and Pymol software was used for 3D protein-binding conformation imaging. The modeling results revealed that many hydrogen bonding forces were formed between both proteins (Fig. 6A and Supplementary Fig. 5), suggesting that *SEMA6A* might form a tight protein complex with *SEC62*. Subsequently, we purified endogenous *SEMA6A* protein from A498 cell lysate and detected the presence of *SEC62* protein (Fig. 6B). Moreover, the reciprocal interaction was validated by IP-Western blot analysis using an anti-*SEC62* antibody (Fig. 6C). To determine whether this interaction could be reconstituted in vitro, we performed a glutathione S-transferase (GST) pull-down assay using GST-*SEC62* and GST as baits and found that GST-tagged *SEC62*, but not GST alone, efficiently pulled down *SEMA6A* protein, suggesting a direct interaction between *SEC62* and *SEMA6A* (Fig. 6D). Reportedly, *SEC62* has competitively disrupted the interaction between β-catenin and APC to inhibit the β-catenin destruction complex assembly, driving us to assess whether *SEMA6A* affected the interaction between *SEC62* and β-catenin. To test this hypothesis, we purified the *SEC62* protein complex from A498 cells with or without *SEMA6A* depletion and then detected the presence of β-catenin by immunoblotting. Consistent with previous findings, *SEC62* was bound to β-catenin in A498 cells. However, in the absence of *SEMA6A*, the interaction between *SEC62* and β-catenin was impaired (Fig. 6E). On the contrary, overexpression of exogenous *SEMA6A* significantly enhanced this interaction (Fig. 6F). Together, these results indicated that *SEMA6A* promoted the interaction between *SEC62* and β-catenin to prevent the degradation of β-catenin protein, thereby indicating that *SEC62* might be critical for *SEMA6A*-induced β-catenin activation and biological functions. Luciferase assay showed that *SEC62* knockdown decreased the transcriptional ability of β-catenin induced by *SEMA6A* (Fig. 6G). Furthermore, *SEC62* depletion significantly reversed *SEMA6A*-induced A498 cell proliferation (Fig. 6H), BrdU incorporation (Fig. 6I), Caspase3/7 activation (Fig. 6J), colony formation (Fig. 6K) and invasion in vitro (Fig. 6L). Importantly, *SEC62* depletion also significantly reversed *SEMA6A*-induced A498 cell proliferation in nude mice in vivo (Fig. 6M–O). Together, our results demonstrated that *HIF-2α*-induced *SEMA6A* expression promoted ccRCC progression through *SEC62*-dependent β-catenin stabilization and activation (Fig. 7).

## Discussion

In the present study, we screened the substrates of the *VHL*-*HIF-2α* axis based on the available transcriptome data. Next, we hypothesized that the mRNA levels of potential substrates of the *VHL*-*HIF-2α* axis are decreased in *VHL*-overexpressing cells and increased in *HIF-2α*-deficient cells. Moreover, the mRNA levels of these potential substrates are positively correlated with the activity of the *HIF-2α* signaling pathway. According to these screening criteria, 4 genes were identified, including *SEMA6A* and 3 known canonical target genes of *HIF-2α*. *SEMA6A* is a multifunctional protein that participates in various cellular processes, including vascular development and angiogenesis [29]. *SEMA6A* can form a complex with plexins to regulate the actin cytoskeleton, motility, and cell proliferation and is involved in cancer development [30]. Next, we showed that *SEMA6A* was elevated under physical hypoxia or pharmacologically-simulated hypoxia conditions, whereas genetic ablation or pharmacological inhibition of *HIF-2α* significantly decreased *SEMA6A* expression in ccRCC cells, suggesting that hypoxia-induced *SEMA6A* expression requires the transcriptional activity of *HIF-2α*. Typically, ChIP and luciferase assays revealed that *HIF-2α* directly activates *SEMA6A* transcription in hypoxic ccRCC cells. The pooled TCGA and GEO data showed that *SEMA6A* mRNA was significantly increased in ccRCC compared to normal tissues and benign kidney tissues, suggesting that *SEMA6A* is involved in ccRCC progression. Consistent with this finding, depletion of *SEMA6A* significantly restrained the proliferation and migration ability of ccRCC in vitro and limited the in vivo growth of cancer in nude mice. Together, these results highlighted an oncogenic role of *SEMA6A* in ccRCC.

The transcriptome data analysis of *SEMA6A* KO mice tissue showed that the *Wnt*/β-catenin pathway is significantly downregulated in case of *SEMA6A* depletion. TCGA data analysis demonstrated that the mRNA expression of *SEMA6A* is closely correlated to the activation of *Wnt*/β-catenin pathway in ccRCC. The *Wnt*/β-catenin pathway is implicated in numerous signaling pathways, including embryogenesis, cell proliferation, migration and invasion, apoptosis, and organogenesis [31]. The abnormal activation of the signaling pathway induces tumorigenesis [32]. The current results showed that depletion of *SEMA6A* decreases the *Wnt*/β-catenin activity, as shown by impaired TOPflash luciferase activity and reduced *Wnt*/β-catenin downstream target gene expression. Conversely, the overexpression of *SEMA6A* activated the *Wnt*/β-catenin pathway, which could be reversed by pharmacological β-catenin inhibition. Also, β-catenin is a multifunctional protein encoded by the *CTNNB1* gene that mediates signal transduction and exerts a core role in promoting tumor proliferation and metastasis in various malignancies [33]. Although *SEMA6A* knockdown inhibited the activity of the *Wnt*/β-catenin pathway, the mRNA level of *β-catenin* was not affected. Interestingly, the protein level of β-catenin is downregulated in *SEMA6A*-depleted cells, suggesting that *SEMA6A* regulates β-catenin at the posttranscriptional level. Consistent with this phenomenon, we showed that decreased β-catenin protein level in *SEMA6A*-depleted cells was caused by enhanced ubiquitination and degradation. The turnover of β-catenin is critical for the inactivation of the *Wnt*/β-catenin signaling pathway [34]. Reportedly, *SEC62*, a component of the protein translocation tool on the endoplasmic reticulum membrane, competitively disrupted the interaction between β-catenin and APC to prevent β-catenin destruction [28]. Interestingly, *SEC62* is found to be a putative interaction protein of *SEMA6A*. Several technical methods at different levels confirmed the interaction between *SEMA6A* and *SEC62* and found a critical role of *SEMA6A* in *SEC62*-mediated β-catenin stabilization and activation. Thus, our finding elucidated the molecular mechanism through which *SEMA6A* stabilizes β-catenin. We also found that depletion of *SEC62* significantly reverses *SEMA6A*-induced malignant phenotypes in ccRCC both in vitro and in vivo, indicating a critical role of the *SEMA6A*-*SEC62* axis in maintaining the malignant phenotypes of ccRCC.

Collectively, *SEMA6A* is identified as a novel target gene of *VHL*-*HIF-2α* with a cancer-promoting role in ccRCC. *SEMA6A* activates the *Wnt*/β-catenin signaling pathway by inhibiting the β-catenin ubiquitination and degradation, suggesting that *SEMA6A* acts as a potential therapeutic target in ccRCCs.

## Supplementary information

Supplementary Figure legends

Supplementary Fig 1

Supplementary Fig 2

Supplementary Fig 3

Supplementary Fig 4

Supplementary Fig 5

Supplementary Fig 6

Supplementary Table1

Supplementary Table2

Supplementary Table3

Supplementary Table4

Supplementary Table5

Supplementary Table6

Supplementary Table7

Supplementary Table8

Original Data File

Reproducibility checklist

## Data Availability

All data generated or analyzed in this study are included in this paper and can be obtained from the corresponding author according to formal requirement.
