# Supplementary material for: VHL-HIF-2α axis-induced SEMA6A upregulation stabilized β-catenin to drive clear cell renal cell carcinoma progression
Source: Cell Death Dis. 2023 Feb 4;14(2):83. doi: 10.1038/s41419-023-05588-4 (PMC9899268; doi:10.1038/s41419-023-05588-4)
Supplement: Supplementary file 1 — Supplementary Figure legends [file 41419_2023_5588_MOESM1_ESM.docx]

**Supplementary Figure legends**

**Figure S1 Hypoxia and hypoxia mimics induced *SEMA6A* expression in 786-O cells.**

A-B Relative mRNA and protein levels of *SEMA6A* in 786-O cells under either normoxia or hypoxia conditions. ***P* < 0.01.

C-D Relative mRNA and protein levels of *SEMA6A* in 786-O cells in response to CoCl_2_ treatment. **P* < 0.05.

**Figure S2 Knockdown of *SEMA6A* inhibited the malignant phenotype of 786-O cells**

1. Western blot analysis of *SEMA6A* protein in 786-O cells stably expressing indicated shRNAs.
2. Proliferation ability of 786-O cells from (A) was determined by CCK-8 assay. ***P* < 0.01.
3. Relative number of BrdU-incorporated cells in 786-O cells from (A). ***P* < 0.01. ****P* < 0.001.
4. Relative Caspase3/7 activity in 786-O cells from (A) *p<0.05.
5. Colonies of 786-O cells from (A). ***P* < 0.01, ****P* < 0.001.
6. Invasion ability of 786-O cells from (A). **P* < 0.05, ***P* < 0.01.

**Figure S3 Knockdown of *SEMA6A* inhibited the malignant phenotype of Caki-1 cells**

1. Western blot analysis of *SEMA6A* protein in Caki-1 cells stably expressing indicated shRNAs.
2. Proliferation ability of Caki-1 cells from (A) was determined by CCK-8 assay. *p<0.05, ***P* < 0.01.
3. Relative number of BrdU-incorporated cells in Caki-1 cells from (A). ***P* < 0.01.
4. Relative Caspase3/7 activity in Caki-1 cells from (A) *p<0.05.
5. Colonies of Caki-1 cells from (A). ***P* < 0.01,
6. Invasion ability of Caki-1 cells from (A). ***P* < 0.01.

**Figure S4 Interaction protein network of *SEMA6A* revealed by the BioGRID database (https://thebiogrid.org/).**

**Figure S5** Structure-based computational analyses revealed many hydrogen bonding forces were formed between *SEMA6A* and *SEC62*. The C chain represents the residues of *SEC62* protein, and the M chain represents the residues of *SEM6A* protein.

**Figure S6 ICG-001 administration significantly delayed *SEMA6A*-induced ccRCC proliferation**

1. A498 cells stably expressing empty vector (EV) or *SEMA6A* were treated with DMSO or ICG-001 as indicated. The proliferation ability of these cells was determined by CCK-8 assay. ****P* < 0.001.
2. Relative Caspase3/7 activity in A498 cells stably expressing EV or *SEMA6A* from (A) ****P* < 0.001.
3. Colonies of A498 cells stably expressing EV or *SEMA6A* from (A). ***P* < 0.01,

**Table S1.List of genes that were up-regulated in the HIF2A_KO group compared with the control group.**

**Table S2. List of genes that were down-regulated in the HIF2A_KO group compared with the control group.**

**Table S3. List of genes that were up-regulated in the HA-VHL reintroduced group compared with the control group.**

**Table S4. List of genes that were down-regulated in the HA-VHL reintroduced group compared with the control group.**

**Table S5. The correlation between SEMA6A and 99 hypoxia metagenes in the TCGA-KIRC database.**

**Table S6. The correlation between SEMA6A and 26 hypoxia metagenes in the TCGA-KIRC database.**

**Table S7. List of genes that were up-regulated in the Sema6A_KO group compared with the control group.**

**Table S8. List of genes that were down-regulated in the Sema6A_KO group compared with the control group.**
