# Supplementary figures and images for: VHL-HIF-2α axis-induced SEMA6A upregulation stabilized β-catenin to drive clear cell renal cell carcinoma progression
Source: Cell Death Dis. 2023 Feb 4;14(2):83. doi: 10.1038/s41419-023-05588-4 (PMC9899268; doi:10.1038/s41419-023-05588-4)

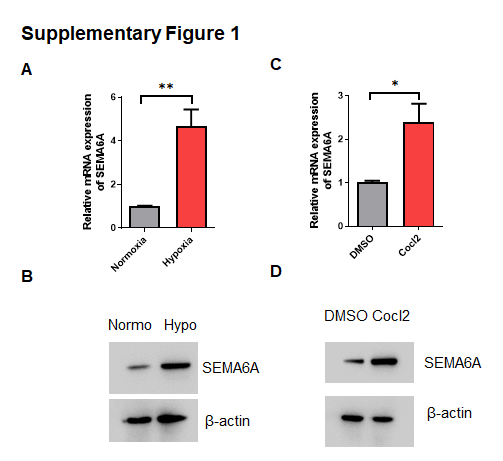

Supplement: Supplementary file 2 — Supplementary Fig 1 [file 41419_2023_5588_MOESM2_ESM.tif]

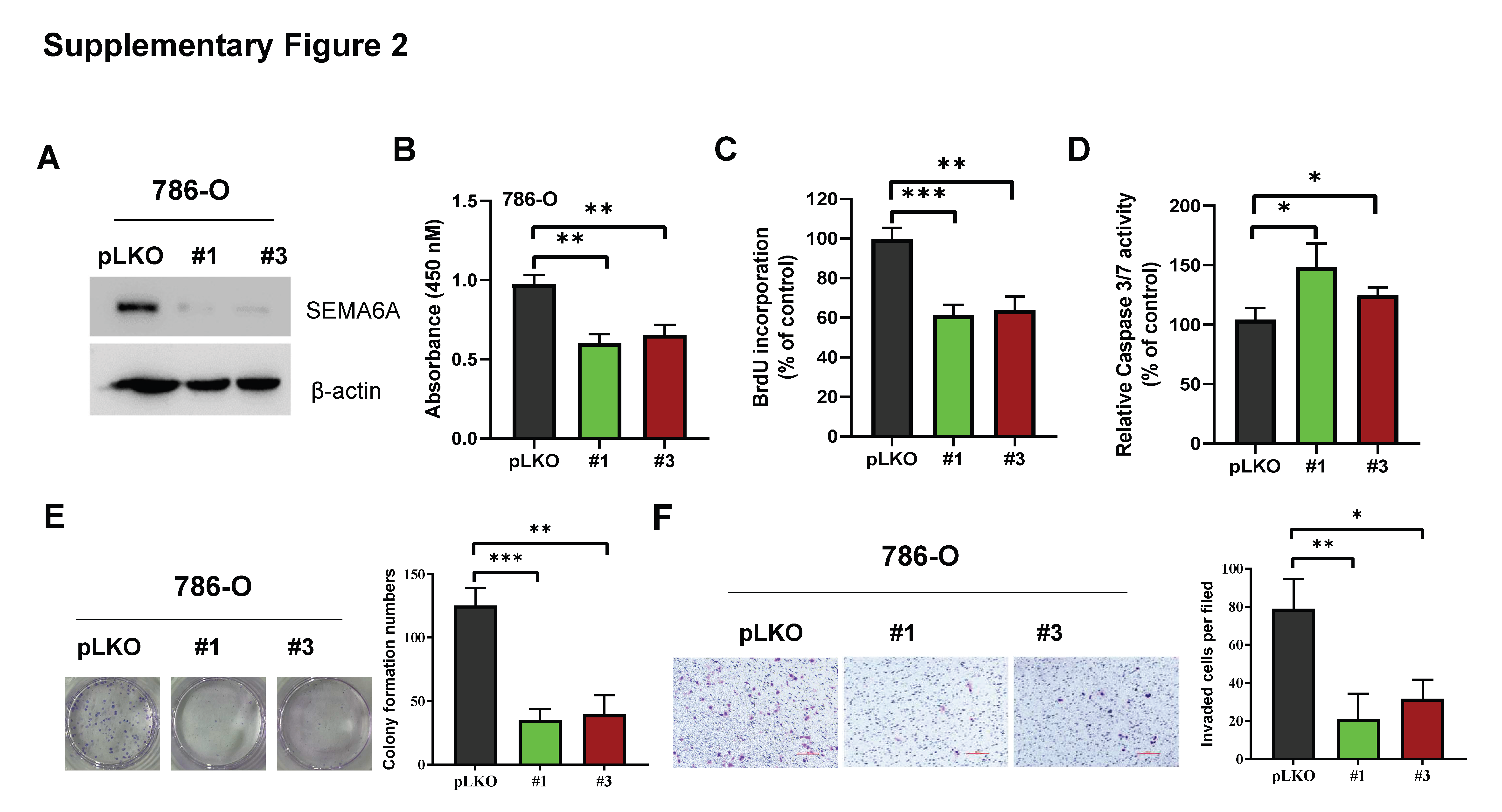

Supplement: Supplementary file 3 — Supplementary Fig 2 [file 41419_2023_5588_MOESM3_ESM.tif]

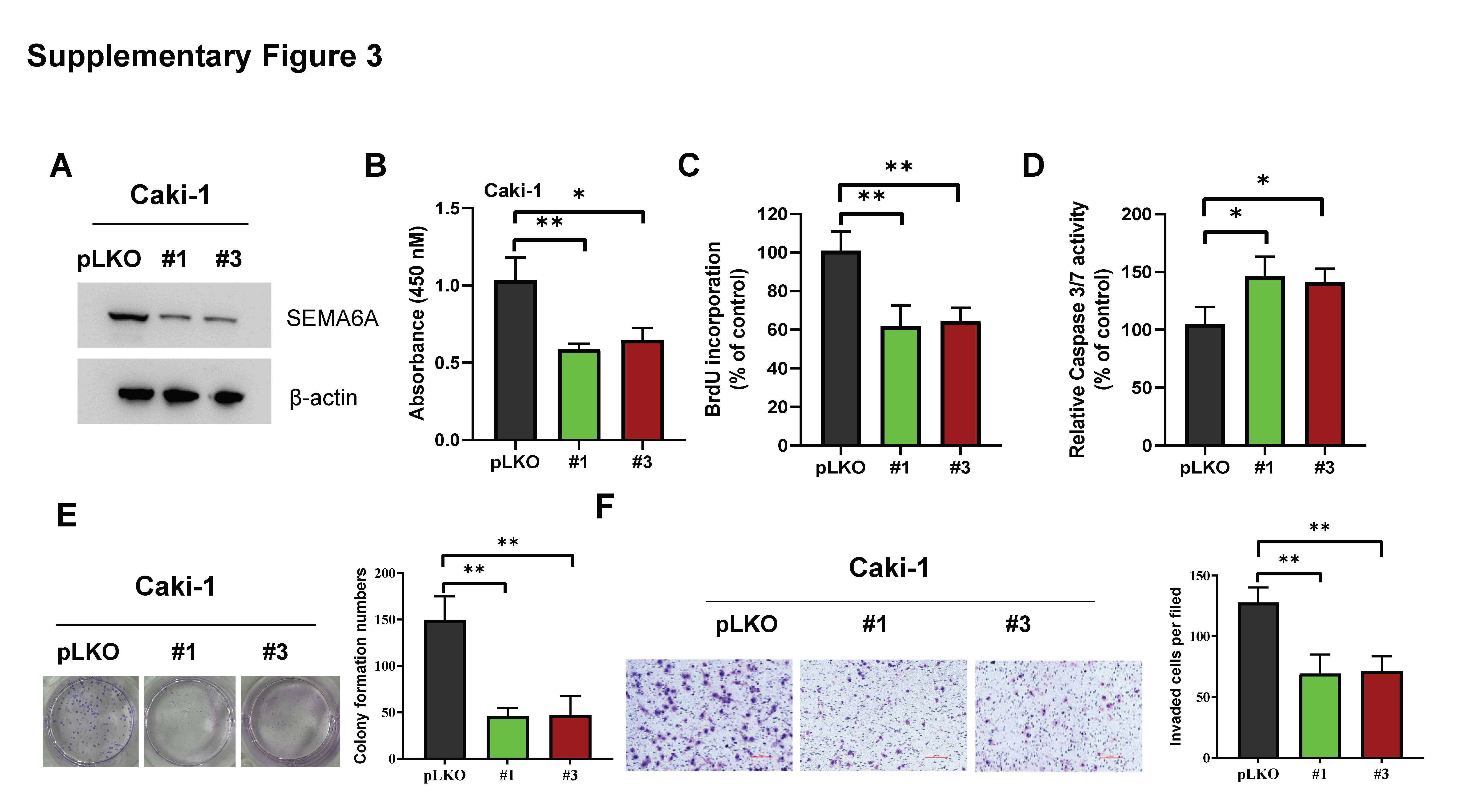

Supplement: Supplementary file 4 — Supplementary Fig 3 [file 41419_2023_5588_MOESM4_ESM.tif]

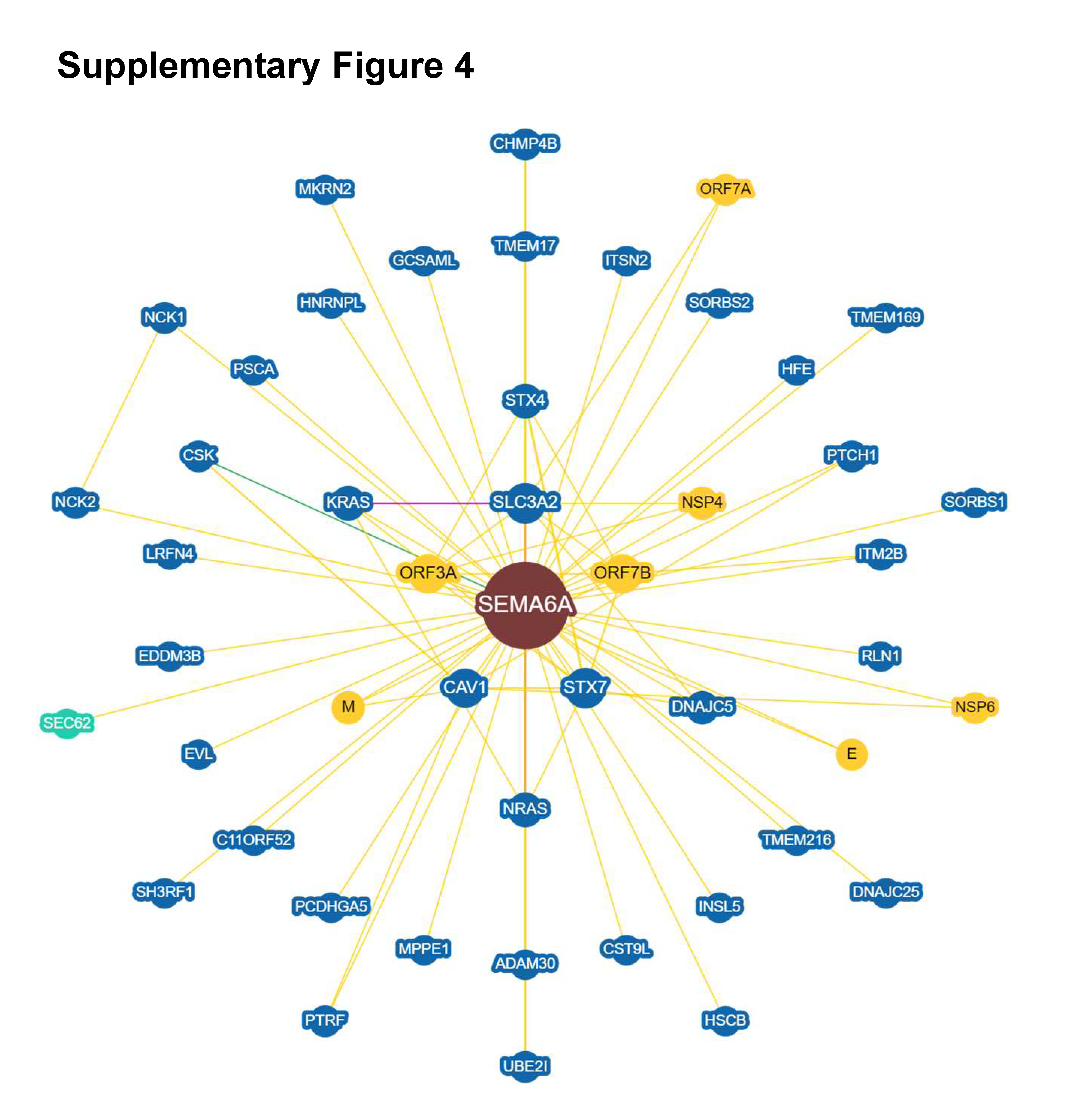

Supplement: Supplementary file 5 — Supplementary Fig 4 [file 41419_2023_5588_MOESM5_ESM.tif]

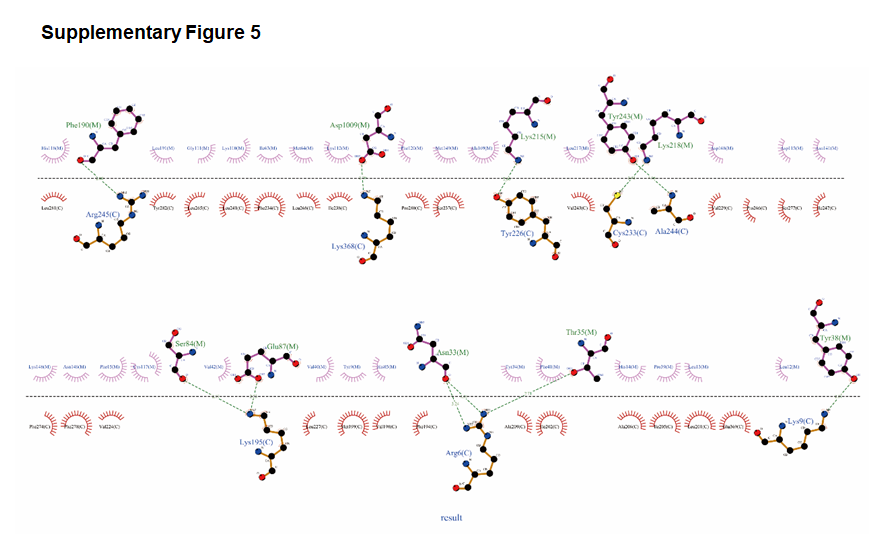

Supplement: Supplementary file 6 — Supplementary Fig 5 [file 41419_2023_5588_MOESM6_ESM.tif]

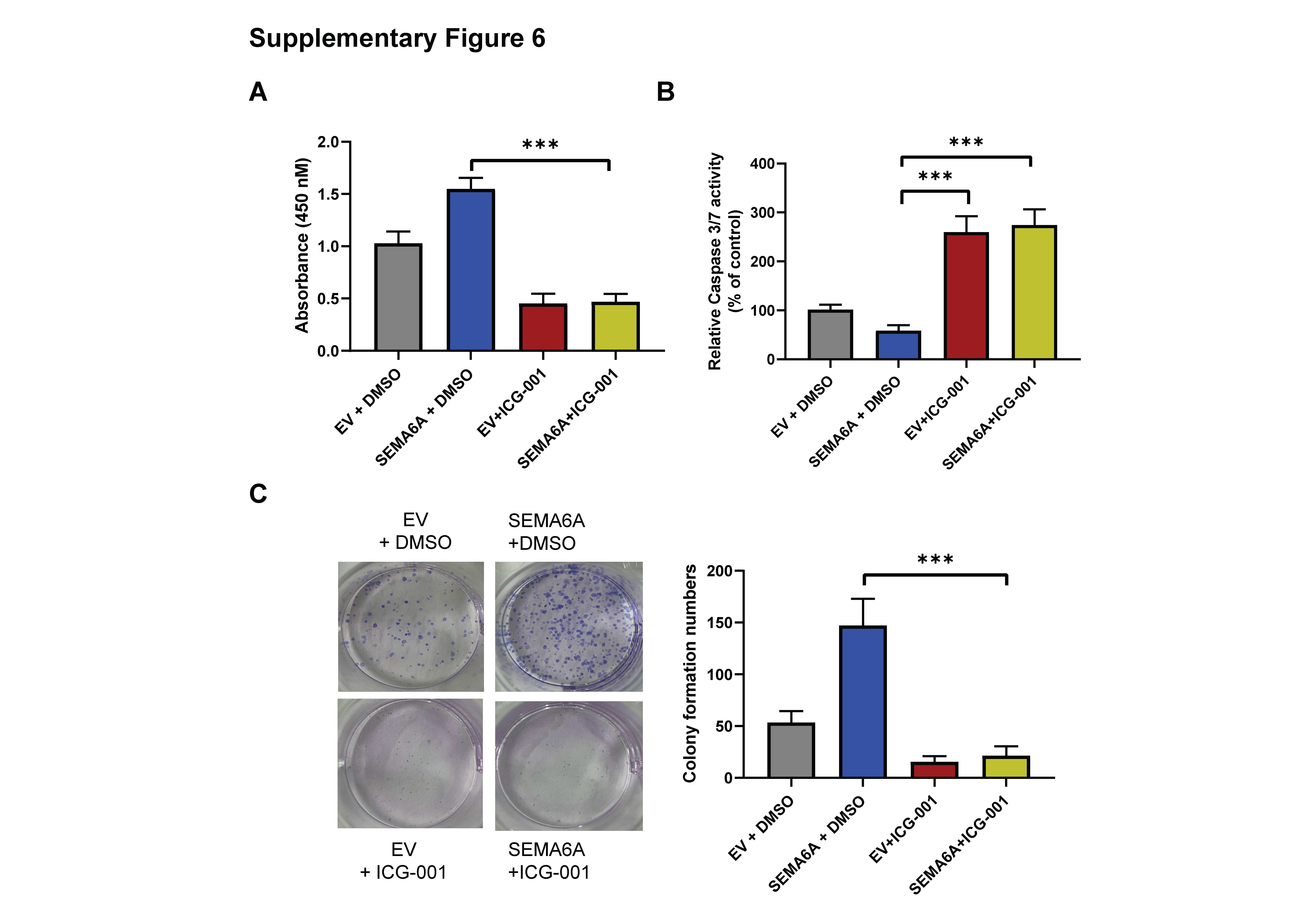

Supplement: Supplementary file 7 — Supplementary Fig 6 [file 41419_2023_5588_MOESM7_ESM.tif]
