# Supplementary material for: VHL-HIF-2α axis-induced SEMA6A upregulation stabilized β-catenin to drive clear cell renal cell carcinoma progression
Source: Cell Death Dis. 2023 Feb 4;14(2):83. doi: 10.1038/s41419-023-05588-4 (PMC9899268; doi:10.1038/s41419-023-05588-4)
Supplement: Supplementary file 8 — Supplementary Table1 [file 41419_2023_5588_MOESM8_ESM.pdf]

Table S1.List of genes that were up-regulated in the HIF2A_KO group compared with the control group.

NOTE:GSE149005, logFC>1.2,P<0.05

Gene	logFC	AveExpr	t	P.Value	adj.P.Val	B	threshold
CLDN2	1.990199	11.16297	39.18919	7.90E-10	5.39E-06	13.57817	Up
MAL2	1.953964	11.25063	37.91255	1.01E-09	5.50E-06	13.33552	Up
SLC7A7	1.670132	10.22668	35.33085	1.69E-09	6.86E-06	12.81293	Up
TSPAN15	2.946147	9.255009	28.54899	8.05E-09	2.40E-05	11.19343	Up
CDH13	1.507991	10.89236	27.83856	9.68E-09	2.40E-05	10.99871	Up
PIGR	2.682101	8.073727	26.8715	1.25E-08	2.45E-05	10.72457	Up
EFHD1	1.359386	7.187289	25.46963	1.85E-08	3.02E-05	10.30723	Up
GPRC5B	1.848687	11.29242	25.42548	1.88E-08	3.02E-05	10.29368	Up
IL18	2.645017	8.185852	25.16541	2.02E-08	3.07E-05	10.21334	Up
AKR1C1	1.912556	7.317	21.64981	6.06E-08	5.17E-05	9.030401	Up
L1CAM	2.374428	8.692703	19.48328	1.30E-07	8.99E-05	8.195622	Up
TRHDE	1.551324	7.449485	19.45412	1.32E-07	8.99E-05	8.18374	Up
SOD3	1.95281	8.317085	18.99646	1.57E-07	9.59E-05	7.994828	Up
SRGN	1.505802	8.779108	18.21281	2.12E-07	0.00011	7.66028	Up
LCP1	1.56565	9.267695	18.20378	2.13E-07	0.00011	7.656338	Up
CERS4	1.278041	6.181864	18.14716	2.18E-07	0.00011	7.631589	Up
ADAMTS12	1.947329	9.783466	17.68706	2.62E-07	0.000118	7.427526	Up
KRT19	1.646096	11.3118	16.88402	3.67E-07	0.000138	7.058184	Up
HCK	1.897952	7.164897	16.47804	4.37E-07	0.000147	6.864709	Up
SLCO2A1	1.390783	6.968276	16.30909	4.71E-07	0.000153	6.78279	Up
BCAM	1.551767	9.54717	16.08145	5.21E-07	0.000165	6.671067	Up
PRKAR2B	1.561796	9.284452	16.02915	5.33E-07	0.000167	6.645179	Up
C3	1.942859	14.59191	15.99888	5.41E-07	0.000168	6.630157	Up
C4orf19	1.850637	8.586267	15.92042	5.60E-07	0.000172	6.591086	Up
SORCS2	1.408849	11.40288	15.84253	5.80E-07	0.000172	6.552111	Up
TRHDE-AS1	1.346485	6.965783	15.84037	5.81E-07	0.000172	6.55103	Up
SLC12A7	1.574475	8.881236	15.83159	5.83E-07	0.000172	6.546621	Up
CSGALNACT	1.920225	7.719647	15.76386	6.01E-07	0.000175	6.512552	Up
CDH4	1.275151	9.263348	15.68213	6.24E-07	0.000179	6.471251	Up
GPRC5A	1.357069	8.184821	15.63859	6.37E-07	0.000181	6.449161	Up
FCAMR	2.395204	10.97052	15.48828	6.82E-07	0.00019	6.372434	Up
PFKFB3	1.492108	11.97691	15.44901	6.95E-07	0.000192	6.352266	Up
HAS3	2.13256	10.75816	15.21649	7.75E-07	0.0002	6.231817	Up
IL4I1	1.796796	9.462089	15.2105	7.77E-07	0.0002	6.228692	Up
ADGRE1	1.333195	6.031704	15.02581	8.48E-07	0.000209	6.131687	Up
SIGLEC15	1.503268	6.964401	14.67939	1.00E-06	0.000238	5.946558	Up
BCO2	1.794694	7.852095	14.59247	1.05E-06	0.000244	5.899441	Up
CXCL12	4.232188	9.939241	14.55944	1.06E-06	0.000245	5.881465	Up
INS-IGF2	2.109064	7.555319	14.27671	1.22E-06	0.00027	5.72595	Up
IGF2	3.147874	9.227908	14.2719	1.23E-06	0.00027	5.723277	Up
TUBB4A	2.099768	7.823559	14.25308	1.24E-06	0.00027	5.712813	Up
F2RL1	1.270702	9.154472	14.07093	1.36E-06	0.000289	5.610872	Up
A4GALT	1.385443	10.0609	14.0228	1.39E-06	0.000292	5.583723	Up
MMP7	1.812061	9.492454	13.86083	1.51E-06	0.000314	5.491697	Up
EPCAM	1.473599	7.291348	13.4054	1.92E-06	0.000341	5.227296	Up
NPTXR	1.560206	10.8746	13.32213	2.00E-06	0.000346	5.17803	Up
ENPP2	1.905438	7.529206	13.30211	2.02E-06	0.000347	5.166142	Up

KLHL13	1.268507	9.660227	13.09913	2.26E-06	0.000355	5.04463	Up
LCN2	1.973976	8.918014	12.79412	2.67E-06	0.000394	4.858636	Up
CCDC8	1.490137	7.661201	12.65791	2.88E-06	0.000416	4.77422	Up
ATP8B1	1.444375	8.279648	12.38886	3.35E-06	0.00046	4.604938	Up
FEZ1	1.740241	8.278207	12.30187	3.52E-06	0.000471	4.549472	Up
KCNJ2	1.65929	7.439624	11.74198	4.89E-06	0.000589	4.18345	Up
CRYM	1.913746	6.569192	10.6768	9.53E-06	0.000936	3.440458	Up
MAL	2.150288	8.450701	10.67295	9.55E-06	0.000936	3.437655	Up
SCN1B	1.524402	7.811795	10.66663	9.59E-06	0.000936	3.433048	Up
PLD5	1.279632	5.995995	10.6435	9.74E-06	0.000939	3.416163	Up
TNFRSF21	1.402901	9.200949	10.36982	1.17E-05	0.001043	3.213869	Up
FZD1	1.213106	10.89869	10.28516	1.24E-05	0.001079	3.150325	Up
LOC10050	1.775004	6.407017	9.999199	1.50E-05	0.001244	2.932205	Up
SLC43A2	1.978102	9.591386	9.966787	1.54E-05	0.001257	2.907136	Up
SAA2	1.799642	11.00353	9.664599	1.90E-05	0.001467	2.669902	Up
CTSF	1.287072	6.19363	9.623102	1.96E-05	0.001494	2.63682	Up
LINC0217	1.390706	7.374849	9.611687	1.97E-05	0.001498	2.627699	Up
ADAMTS15	1.521851	8.681692	9.458105	2.21E-05	0.00162	2.504047	Up
DGAT2	1.364092	8.504952	9.250204	2.57E-05	0.001802	2.33387	Up
PIK3AP1	1.331206	7.498834	9.228634	2.61E-05	0.001824	2.316026	Up
TK2	1.281098	9.651917	9.166737	2.73E-05	0.001881	2.264623	Up
EMP1	1.222453	6.589512	9.069569	2.94E-05	0.001966	2.183329	Up
PPL	1.778736	6.91324	8.79057	3.64E-05	0.002189	1.94573	Up
VWA1	1.214287	7.828191	8.540788	4.42E-05	0.002457	1.727576	Up
MEIS3	1.641285	7.44446	8.371784	5.06E-05	0.002691	1.576946	Up
ADORA2A	1.709132	8.32258	8.345547	5.17E-05	0.002706	1.553338	Up
COL14A1	1.347297	11.0923	8.25341	5.57E-05	0.00283	1.469944	Up
FOXQ1	1.613812	8.870554	7.824221	7.97E-05	0.003623	1.071226	Up
DEPTOR	1.372545	7.687068	7.641772	9.32E-05	0.003967	0.896411	Up
ITGA11	1.366388	6.380103	7.624975	9.46E-05	0.004	0.880152	Up
CGNL1	1.569432	10.31135	7.563356	9.98E-05	0.004125	0.820264	Up
SEMA6B	1.20845	9.542139	7.469764	0.000108	0.004358	0.728568	Up
ADAMTS14	1.215875	6.505856	7.428582	0.000112	0.004457	0.687938	Up
SOCS1	1.27044	7.746075	7.349593	0.000121	0.004611	0.609515	Up
PLLP	1.791023	8.455071	7.348902	0.000121	0.004611	0.608827	Up
SLC16A2	1.623338	10.9667	7.316138	0.000124	0.0047	0.576105	Up
MXRA5	2.760821	8.246782	7.292168	0.000127	0.004759	0.552094	Up
CDH6	1.31083	14.16209	7.165532	0.000142	0.005127	0.424233	Up
STAT4	1.644908	6.846323	7.162359	0.000143	0.00513	0.421008	Up
TMPRSS2	1.640817	9.277025	7.135851	0.000146	0.005227	0.394017	Up
AKR1C2	1.231074	6.040429	6.950435	0.000174	0.005797	0.203084	Up
C14orf13	1.325981	7.821079	6.939799	0.000176	0.00583	0.192017	Up
PLCXD3	1.605101	6.623651	6.887457	0.000184	0.006032	0.137367	Up
TSPAN18	3.1887	8.384313	6.704259	0.000219	0.00679	-0.05635	Up
IGDCC4	1.308206	7.906156	6.696914	0.000221	0.006807	-0.0642	Up
EFEMP1	1.326128	9.184629	6.607194	0.000241	0.00726	-0.16055	Up
SULT1C2	1.717835	6.835091	6.549106	0.000255	0.007542	-0.22344	Up
GDF15	1.767062	10.08314	6.546029	0.000256	0.007556	-0.22679	Up
HMCN1	1.519529	8.807026	6.540833	0.000257	0.007562	-0.23243	Up
SDK1	1.290882	9.278443	6.472821	0.000275	0.007889	-0.30664	Up
TNC	1.599516	11.74911	6.43115	0.000287	0.008102	-0.35238	Up
THSD4	1.202221	8.666165	6.413211	0.000292	0.008205	-0.37214	Up
MYO5B	1.240831	9.300828	6.359439	0.000308	0.008508	-0.43159	Up
SLC48A1	1.411927	9.61562	6.230102	0.000351	0.009359	-0.57603	Up

AKR1B10	1.639821	10.25737	6.092924	0.000403	0.010402	-0.73151	Up
FAM213A	1.67021	7.658956	6.088229	0.000405	0.010433	-0.73687	Up
SAA1	1.599671	12.56977	5.797328	0.00055	0.012706	-1.07468	Up
AK5	1.366999	9.756514	5.770788	0.000566	0.012951	-1.10604	Up
JUP	1.316737	8.591989	5.755215	0.000575	0.013125	-1.12449	Up
SLPI	1.878976	8.227406	5.022782	0.001307	0.022493	-2.02937	Up
HPGD	1.245213	7.118984	4.973687	0.001384	0.023554	-2.09269	Up
CDH11	1.500443	9.389973	4.892446	0.001524	0.02523	-2.19822	Up
DLGAP1	1.41256	7.42435	4.706423	0.001905	0.029453	-2.44341	Up
PLAT	1.293222	7.370282	4.550578	0.002307	0.033712	-2.65262	Up
GPRC5C	1.460701	9.824787	4.267291	0.003295	0.042842	-3.04174	Up
NRCAM	1.393193	8.094619	3.939403	0.005054	0.05691	-3.50602	Up
ITGB4	1.207697	7.103516	3.930565	0.005114	0.057158	-3.51874	Up
GDA	1.464692	6.48521	3.765296	0.006385	0.066541	-3.75834	Up
KRT81	1.259487	6.787709	3.739906	0.006609	0.068041	-3.79545	Up
LZTS1	1.448644	8.118177	3.584291	0.008179	0.078305	-4.02461	Up
BRINP2	1.251605	6.772366	2.563514	0.035765	0.209741	-5.57797	Up
