# Supplementary material for: VHL-HIF-2α axis-induced SEMA6A upregulation stabilized β-catenin to drive clear cell renal cell carcinoma progression
Source: Cell Death Dis. 2023 Feb 4;14(2):83. doi: 10.1038/s41419-023-05588-4 (PMC9899268; doi:10.1038/s41419-023-05588-4)
Supplement: Supplementary file 9 — Supplementary Table2 [file 41419_2023_5588_MOESM9_ESM.pdf]

Table S2.List of genes that were down-regulated in the HIF2A_KO group compared with the control group.

NOTE:GSE149005, logFC<-1.2,P<0.05

Gene	logFC	AveExpr	t	P.Value	adj.P.Val	B	threshold
CPE	-4.44419	8.930759	-42.8035	4.13E-10	3.93E-06	14.21493	Down
CDKN1A	-1.78842	9.744875	-35.1385	1.76E-09	6.86E-06	12.77217	Down
GSTP1	-2.82634	8.971391	-31.6426	3.79E-09	1.29E-05	11.98188	Down
PTHLH	-2.92135	7.40696	-27.8576	9.63E-09	2.40E-05	11.00399	Down
SLC2A1	-1.93366	12.80556	-27.0526	1.19E-08	2.45E-05	10.77672	Down
PLXDC2	-3.19677	7.269596	-25.8807	1.65E-08	3.00E-05	10.43215	Down
ATP1B1	-2.16785	11.31292	-24.9106	2.18E-08	3.13E-05	10.13374	Down
IGFL2-AS1	-1.77549	6.02966	-24.7436	2.29E-08	3.13E-05	10.08111	Down
ADM	-2.16442	9.09882	-24.3529	2.57E-08	3.35E-05	9.956417	Down
C1orf21	-2.02842	6.499247	-23.0413	3.85E-08	4.59E-05	9.521576	Down
IGFBP1	-2.67073	6.867962	-22.9391	3.98E-08	4.59E-05	9.486561	Down
PTGS2	-1.49021	6.356256	-22.7042	4.29E-08	4.59E-05	9.405532	Down
SLCO2B1	-1.32227	9.383516	-22.5932	4.44E-08	4.59E-05	9.366918	Down
LUCAT1	-1.63848	6.469472	-22.5919	4.44E-08	4.59E-05	9.366451	Down
MGMT	-2.00187	6.157858	-22.5279	4.54E-08	4.59E-05	9.344102	Down
CLDN4	-1.9164	7.176854	-22.1912	5.06E-08	4.94E-05	9.225361	Down
BHLHE40	-1.70706	10.66263	-21.9101	5.55E-08	5.11E-05	9.124766	Down
LINC01929	-1.93326	6.338835	-21.8714	5.63E-08	5.11E-05	9.110809	Down
MYH13	-3.25183	6.328508	-21.7792	5.80E-08	5.11E-05	9.077446	Down
GAL3ST1	-3.45468	10.06249	-21.2054	7.05E-08	5.83E-05	8.866481	Down
SLITRK5	-1.78258	6.140445	-20.7625	8.21E-08	6.60E-05	8.699522	Down
NIPAL2	-1.55527	7.47758	-20.338	9.54E-08	7.45E-05	8.535963	Down
SEMA5B	-2.63572	8.556494	-19.8786	1.13E-07	8.31E-05	8.354908	Down
SEMA6A	-3.66911	7.870303	-19.7872	1.16E-07	8.37E-05	8.318386	Down
EGR1	-1.81928	7.087107	-19.364	1.36E-07	9.08E-05	8.146884	Down
PCDH10	-1.3123	5.689501	-18.9129	1.62E-07	9.59E-05	7.959849	Down
MAGEA12	-1.89617	6.408446	-18.4488	1.93E-07	0.000108	7.762543	Down
ENPP3	-3.65556	7.362807	-18.1328	2.19E-07	0.00011	7.625294	Down
LINC01296	-1.54497	6.698175	-17.8119	2.49E-07	0.000118	7.483426	Down
PTGES	-2.03405	6.985873	-17.7771	2.53E-07	0.000118	7.467865	Down
TGFA	-2.42086	10.88372	-17.6143	2.70E-07	0.000118	7.39475	Down
SMPDL3A	-2.80083	7.59629	-17.2778	3.11E-07	0.000127	7.241444	Down
DOCK2	-1.29483	6.8658	-17.0316	3.45E-07	0.000134	7.127375	Down
SLC6A9	-1.76296	6.232881	-17.0024	3.49E-07	0.000134	7.113749	Down
ARTN	-1.63841	7.174677	-16.8522	3.72E-07	0.000138	7.043205	Down
CADM1	-1.33113	10.45918	-16.6131	4.12E-07	0.000147	6.929599	Down
DGCR5	-1.48017	6.206657	-16.4967	4.34E-07	0.000147	6.873728	Down
IGFBP3	-1.91027	13.99862	-16.4822	4.36E-07	0.000147	6.866737	Down
MDGA2	-1.66124	5.893604	-16.4781	4.37E-07	0.000147	6.864758	Down
TMEM47	-3.7201	7.194477	-16.4101	4.50E-07	0.00015	6.831865	Down
EDA2R	-2.24429	6.424892	-16.3265	4.67E-07	0.000153	6.791279	Down
LOC613266	-2.57371	7.522421	-15.818	5.87E-07	0.000172	6.539775	Down
C10orf10	-2.36331	8.828469	-15.2858	7.50E-07	0.0002	6.267898	Down
MAP7D2	-2.72861	7.689577	-15.213	7.76E-07	0.0002	6.229975	Down
LIN7A	-1.50169	5.704232	-15.2097	7.77E-07	0.0002	6.228288	Down
CCND1	-1.63773	12.30647	-15.0205	8.50E-07	0.000209	6.128859	Down
MYEOV	-3.37984	6.758081	-14.9307	8.88E-07	0.000216	6.081257	Down
TNFAIP6	-1.36484	6.561654	-14.887	9.06E-07	0.000219	6.057986	Down

XAF1	-1.35059	5.870913	-14.8551	9.20E-07	0.000221	6.040969	Down
ACKR3	-3.1178	6.599539	-14.2432	1.24E-06	0.00027	5.707336	Down
AFF2	-2.431	6.367776	-14.1452	1.31E-06	0.000281	5.652613	Down
RNASET2	-2.4602	11.00823	-13.8533	1.52E-06	0.000314	5.487386	Down
FZD8	-1.67741	7.852964	-13.7717	1.58E-06	0.000318	5.440604	Down
PLSCR4	-1.47641	6.01661	-13.6988	1.64E-06	0.000323	5.398625	Down
CYTIP	-1.25058	5.661376	-13.6607	1.67E-06	0.000323	5.376567	Down
MACROD2	-3.04675	7.768662	-13.4402	1.88E-06	0.00034	5.247815	Down
SLITRK2	-1.84882	5.885662	-13.2663	2.06E-06	0.000348	5.144831	Down
MIR34AHG	-1.35607	6.361054	-13.1728	2.17E-06	0.000354	5.088952	Down
LAYN	-1.73437	5.949234	-13.1373	2.21E-06	0.000355	5.067584	Down
PLAC8	-1.48408	7.683558	-13.096	2.26E-06	0.000355	5.04275	Down
EGLN3	-3.71541	8.020365	-12.7285	2.77E-06	0.000404	4.818071	Down
PLEKHA7	-1.20419	7.000995	-12.7051	2.80E-06	0.000407	4.803542	Down
IRS2	-3.17209	10.3686	-12.5177	3.12E-06	0.000443	4.686455	Down
GALNT5	-1.91354	6.136333	-12.4894	3.17E-06	0.000448	4.66862	Down
L3MBTL4	-1.40772	5.841664	-12.4613	3.22E-06	0.000451	4.650875	Down
HOST2	-1.29625	7.875232	-12.4247	3.28E-06	0.000455	4.627671	Down
MAP3K21	-1.5984	8.967025	-12.1949	3.75E-06	0.00049	4.480745	Down
ALPK3	-2.22397	7.11755	-12.0954	3.97E-06	0.000509	4.416308	Down
MLLT11	-1.62133	8.323495	-12.0586	4.06E-06	0.000515	4.392408	Down
CP	-1.52163	11.46219	-11.9175	4.41E-06	0.000551	4.299883	Down
TENM2	-1.93919	7.28331	-11.9151	4.41E-06	0.000551	4.298317	Down
TCEA3	-1.38247	6.320498	-11.9001	4.45E-06	0.000552	4.288448	Down
STAC	-1.2677	10.1433	-11.8758	4.52E-06	0.000556	4.272383	Down
DBH-AS1	-1.32365	6.521889	-11.8273	4.65E-06	0.000566	4.240284	Down
LUM	-1.67783	6.325906	-11.704	5.01E-06	0.000592	4.158033	Down
RARRES2	-3.17431	8.621487	-11.5403	5.53E-06	0.000627	4.047672	Down
PTCHD4	-2.22982	6.310333	-11.4777	5.74E-06	0.000637	4.005053	Down
ITPR1	-1.6994	8.002671	-11.277	6.50E-06	0.000698	3.866975	Down
APOL1	-1.46431	13.19834	-11.1658	6.97E-06	0.000732	3.789554	Down
SYNPO	-2.12014	12.60398	-11.1462	7.05E-06	0.000738	3.775855	Down
WDR72	-1.83241	6.008082	-11.0991	7.27E-06	0.000757	3.742778	Down
SARDH	-1.63749	7.976038	-10.8651	8.43E-06	0.00086	3.576591	Down
BMPR1B	-1.73179	6.506649	-10.7268	9.22E-06	0.000916	3.476806	Down
NDRG1	-3.28335	11.73851	-10.5882	1.01E-05	0.000961	3.375669	Down
LAMA2	-1.91594	7.32104	-10.4837	1.08E-05	0.001012	3.298639	Down
ZMAT3	-1.39532	8.444519	-10.4565	1.10E-05	0.001019	3.278429	Down
EDN2	-1.49469	6.011399	-10.4421	1.11E-05	0.00102	3.267736	Down
LOC100507	-1.214	6.401348	-10.3666	1.17E-05	0.001043	3.211448	Down
SFRP2	-1.40803	5.928489	-9.87629	1.64E-05	0.001304	2.836763	Down
PRIMA1	-1.325	5.584574	-9.57921	2.02E-05	0.001521	2.601696	Down
MSMO1	-1.41696	10.41808	-9.44695	2.22E-05	0.001628	2.495	Down
ARRDC3	-1.94257	10.56535	-9.4352	2.24E-05	0.001634	2.48546	Down
ITGA4	-1.89117	7.361993	-9.21288	2.64E-05	0.001836	2.302974	Down
KALRN	-1.66563	8.017213	-9.19905	2.67E-05	0.00185	2.291499	Down
ITGB8	-1.83359	11.49003	-8.99056	3.12E-05	0.00202	2.116682	Down
ERRFI1	-1.75744	11.25921	-8.97793	3.15E-05	0.002027	2.105974	Down
ZNF395	-1.78828	10.3097	-8.89682	3.35E-05	0.002092	2.036953	Down
FLNC	-1.29594	12.21693	-8.77867	3.67E-05	0.002199	1.935457	Down
DOCK2	-1.29483	6.8658	-17.0316	3.45E-07	0.000134	7.127375	Down
SLC6A9	-1.76296	6.232881	-17.0024	3.49E-07	0.000134	7.113749	Down
ARTN	-1.63841	7.174677	-16.8522	3.72E-07	0.000138	7.043205	Down
CADM1	-1.33113	10.45918	-16.6131	4.12E-07	0.000147	6.929599	Down

DGCR5	-1.48017	6.206657	-16.4967	4.34E-07	0.000147	6.873728	Down
TTLL7	-1.41543	6.827302	-8.51439	4.52E-05	0.002488	1.704215	Down
CA12	-1.42146	11.76571	-8.48363	4.63E-05	0.002529	1.676913	Down
BHMT2	-1.55778	7.694835	-8.48338	4.63E-05	0.002529	1.676688	Down
PODXL2	-1.50413	6.048984	-8.47976	4.64E-05	0.002532	1.673474	Down
SYK	-1.99538	6.854452	-8.45117	4.75E-05	0.002575	1.648014	Down
TGFBI	-1.61173	15.22167	-8.40818	4.92E-05	0.002639	1.609592	Down
ROR2	-1.40925	8.49148	-8.35162	5.15E-05	0.002703	1.558807	Down
SLAMF8	-1.49164	6.520511	-8.24955	5.59E-05	0.002833	1.46643	Down
ALDH2	-1.47657	7.406023	-8.11701	6.23E-05	0.003046	1.345081	Down
FLG	-4.04643	9.374374	-8.05251	6.58E-05	0.003146	1.285449	Down
LRRK2	-1.36333	6.430014	-8.02468	6.73E-05	0.003192	1.259589	Down
SLC6A6	-1.79734	11.54445	-8.00287	6.85E-05	0.003242	1.239287	Down
POU5F1	-1.50156	7.443255	-7.83393	7.90E-05	0.003611	1.080439	Down
PREX2	-1.8152	7.969041	-7.7765	8.30E-05	0.003717	1.025814	Down
F3	-1.62188	7.96611	-7.76198	8.40E-05	0.003742	1.011958	Down
PCDH7	-1.82854	7.517404	-7.66138	9.17E-05	0.003933	0.915353	Down
TSPAN2	-1.29934	8.956255	-7.64857	9.27E-05	0.003956	0.902982	Down
SLC7A5	-1.92982	11.662	-7.60059	9.66E-05	0.004051	0.856493	Down
VEGFA	-1.31952	12.31246	-7.53263	0.000103	0.004189	0.790259	Down
CFH	-1.99749	9.422636	-7.37409	0.000118	0.00458	0.63391	Down
EPAS1	-1.97161	10.59906	-7.12906	0.000147	0.005239	0.387093	Down
DSP	-2.76754	7.173173	-7.09939	0.000151	0.00532	0.356768	Down
SERPINE1	-3.03353	10.85799	-7.06148	0.000157	0.00542	0.317886	Down
IGFBP5	-1.86422	8.040354	-6.90384	0.000182	0.005961	0.154506	Down
EDN1	-1.48004	6.927407	-6.87984	0.000186	0.006058	0.129388	Down
IFI6	-2.04206	10.01244	-6.84999	0.000191	0.006182	0.098061	Down
MDK	-1.34689	6.181648	-6.81565	0.000197	0.006319	0.061887	Down
PDGFB	-1.31257	8.675216	-6.74732	0.000211	0.006635	-0.01047	Down
CFI	-2.15654	8.069496	-6.51075	0.000265	0.007714	-0.26519	Down
RAB36	-1.33657	6.116558	-6.05849	0.000418	0.010678	-0.7709	Down
CHST9	-1.62938	6.82558	-5.99427	0.000447	0.01114	-0.84479	Down
ALDH1A1	-2.09878	10.48547	-5.97482	0.000456	0.011269	-0.86726	Down
RSAD2	-1.38722	6.023009	-5.89782	0.000494	0.011874	-0.95675	Down
CSAG1	-1.23334	6.028973	-5.65556	0.00064	0.013961	-1.24332	Down
SULF2	-1.3238	7.204329	-5.5136	0.000748	0.015397	-1.41487	Down
ENPEP	-1.73846	9.351446	-5.14789	0.00113	0.020463	-1.86955	Down
S100A2	-1.31934	8.486188	-5.04662	0.001271	0.022121	-1.99875	Down
DDIT4	-1.51359	11.5797	-4.36989	0.002892	0.039273	-2.8995	Down
TRIB3	-1.34979	10.1998	-4.11768	0.003998	0.049098	-3.25177	Down
INSIG1	-1.30336	10.76056	-3.89549	0.005359	0.059097	-3.5693	Down
TXNIP	-1.25439	9.063815	-3.74754	0.00654	0.067543	-3.78428	Down
