# Supplementary material for: VHL-HIF-2α axis-induced SEMA6A upregulation stabilized β-catenin to drive clear cell renal cell carcinoma progression
Source: Cell Death Dis. 2023 Feb 4;14(2):83. doi: 10.1038/s41419-023-05588-4 (PMC9899268; doi:10.1038/s41419-023-05588-4)
Supplement: Supplementary file 10 — Supplementary Table3 [file 41419_2023_5588_MOESM10_ESM.pdf]

Table S3.List of genes that were up-regulated in the HA-VHL reintroduced group compared with the control group.

NOTE:GSE32297, logFC>1.2,P<0.05

Gene	logFC	AveExpr	t	P.Value	adj.P.Val	B	threshold
IL1R1	1.595721756	7.036578017	43.94256653	2.87E-07	0.000455435	7.534707813	Up
MAL2	2.942174554	10.65294622	39.45047619	4.73E-07	0.000545762	7.203635747	Up
SDC2	2.423913553	7.683481649	38.5285453	5.28E-07	0.000545762	7.126762067	Up
DPP4	1.201806034	10.20881179	31.8773451	1.27E-06	0.000823245	6.457377787	Up
ARSE	1.930293383	5.344461043	31.86115015	1.27E-06	0.000823245	6.455460183	Up
ALDH1A1	2.371102155	10.16270436	29.93317511	1.70E-06	0.001023218	6.215207793	Up
ASS1	1.925179439	10.66343545	29.43923151	1.84E-06	0.00106827	6.149621408	Up
LOC440338	3.372370619	9.110000307	28.42995264	2.16E-06	0.001107661	6.010077195	Up
TUBA1A	1.260929183	9.179620197	28.17740271	2.25E-06	0.001117132	5.973949122	Up
MYLIP	1.282032524	4.158898368	26.14472185	3.18E-06	0.001422547	5.664078341	Up
PGBD5	1.833943724	4.523780551	25.31805335	3.69E-06	0.001427215	5.527573449	Up
CDH6	1.399492882	8.784058413	24.85471215	4.02E-06	0.001427215	5.44818502	Up
ELF3	1.847083059	7.948660133	24.6524398	4.17E-06	0.001427215	5.412854316	Up
NAPE-PLD	1.429799995	6.770798686	24.30571869	4.45E-06	0.001427215	5.351315267	Up
MMP7	5.766060254	5.688054125	23.44387445	5.26E-06	0.001585632	5.192794575	Up
FAM84B	1.236604927	7.447975539	22.85503404	5.92E-06	0.001682224	5.079708954	Up
SYTL2	1.806072334	3.985004083	22.27848306	6.65E-06	0.001722908	4.965010962	Up
CRYAB	6.856074806	5.698202688	21.94765522	7.13E-06	0.001728147	4.897346508	Up
IMPA2	1.735423475	9.422565511	21.50822771	7.83E-06	0.001845722	4.805302897	Up
EDG1	2.292231095	8.000071486	21.38143701	8.04E-06	0.001871412	4.778273347	Up
TNFRSF11B	2.324776701	4.20975643	19.93420072	1.11E-05	0.002215513	4.453863731	Up
QPRT	2.022881332	7.767718826	19.08502924	1.36E-05	0.002488628	4.248878493	Up
GPRC5B	1.470221473	7.052693792	18.97359253	1.39E-05	0.002488628	4.221113151	Up
SPP1	1.663573429	12.1440604	18.81204025	1.45E-05	0.002510567	4.180491774	Up
PDZK1	2.402022044	8.512046817	18.36658236	1.62E-05	0.002690095	4.066165919	Up
TNFRSF21	1.332946001	8.307616205	18.19141713	1.69E-05	0.002758611	4.020253938	Up
DCDC2	1.886898011	9.584474373	16.57351735	2.59E-05	0.003535433	3.568651469	Up
KLHL13	4.135421664	5.855620917	15.95734901	3.08E-05	0.00388177	3.382438886	Up
EDG3	1.210455933	7.884295691	15.14015724	3.92E-05	0.004207897	3.121957596	Up
NRCAM	1.255967261	4.287514573	14.80739833	4.34E-05	0.004392862	3.011166742	Up
PCSK5	1.464734218	4.11292642	14.73155829	4.44E-05	0.00445782	2.985514871	Up
LOC440338	3.372370619	9.110000307	28.42995264	2.16E-06	0.001107661	6.010077195	Up
TUBA1A	1.260929183	9.179620197	28.17740271	2.25E-06	0.001117132	5.973949122	Up
MYLIP	1.282032524	4.158898368	26.14472185	3.18E-06	0.001422547	5.664078341	Up
PGBD5	1.833943724	4.523780551	25.31805335	3.69E-06	0.001427215	5.527573449	Up
CDH6	1.399492882	8.784058413	24.85471215	4.02E-06	0.001427215	5.44818502	Up
ELF3	1.847083059	7.948660133	24.6524398	4.17E-06	0.001427215	5.412854316	Up
RARRES1	1.842447894	3.87633977	14.60861415	4.62E-05	0.004601178	2.943607467	Up
SLC16A4	1.46867657	8.007949286	14.21094658	5.24E-05	0.004861788	2.805251165	Up
IL18	2.221091724	7.127376813	13.75296484	6.08E-05	0.005278499	2.640381313	Up
SLC7A7	1.70894399	9.182218249	13.34888423	6.96E-05	0.005659507	2.489732727	Up
ST3GAL5	1.659245315	6.494713978	12.62493328	8.97E-05	0.006647199	2.206810703	Up
SLC22A18	1.513552935	6.481901409	12.60370709	9.04E-05	0.006656461	2.19825006	Up
LUM	3.431126118	4.890719916	12.41501578	9.68E-05	0.006839038	2.121456487	Up
PTPRD	1.241064505	7.196782878	12.39347898	9.76E-05	0.006839045	2.11261134	Up
C4ORF18	1.683382942	4.041723111	12.22508428	0.000104	0.00706416	2.042875805	Up
CLEC4E	1.759479363	9.722560317	12.16856395	0.000106	0.007142277	2.019238052	Up
RAB31	1.814933375	3.124697667	11.92588199	0.000116	0.007304605	1.916391932	Up
OLFML2B	1.728967305	5.02158834	11.41507815	0.000142	0.008177046	1.692462841	Up
FAM49A	2.623389718	7.112812272	11.31381976	0.000147	0.008401085	1.646820829	Up
NID2	1.489341935	6.531776712	11.17932387	0.000155	0.0085776	1.585532512	Up
MMP1	2.593668935	6.783284343	11.14566377	0.000158	0.008594454	1.570073713	Up
GDF15	3.099571676	6.670789513	11.11374005	0.00016	0.008605105	1.555367431	Up

TBC1D8	1.399632776	3.037209254	10.41860102	0.000213	0.010012114	1.223852587	Up
ITGB6	1.90153999	3.455717449	10.36378257	0.000219	0.010115838	1.196753054	Up
LY96	1.430137072	4.87936949	9.422915682	0.000334	0.01261966	0.707749648	Up
SCD	1.933641863	8.715956875	9.195126656	0.000373	0.013303154	0.582089885	Up
AKR1C3	1.576520019	3.932173821	9.151884476	0.000381	0.01346256	0.557892621	Up
ACSL5	1.220312847	5.301864148	8.972526059	0.000416	0.014192612	0.45632929	Up
C10ORF11	1.912816491	2.761136974	8.874351497	0.000436	0.014367193	0.399903262	Up
SLC44A2	1.235220071	5.65858969	8.731849344	0.000469	0.015127372	0.316923674	Up
GPR160	2.458131171	7.672195389	8.730885699	0.000469	0.015127372	0.316358138	Up
TCEA3	2.28957598	4.034966549	8.688551092	0.000479	0.015186623	0.29145414	Up
KCNJ16	2.167015144	5.535677077	8.561913895	0.000511	0.015818409	0.216261704	Up
RNF144B	1.652060045	3.421328696	8.375622666	0.000563	0.016632444	0.10371073	Up
FBXO6	1.434720693	6.558015619	8.086500622	0.000657	0.018411321	-0.07571099	Up
FRMD4B	1.588588087	7.277330854	7.855740754	0.000746	0.020031059	-0.22324255	Up
KLHL14	1.68964421	3.76705139	7.826449162	0.000758	0.020267166	-0.24225403	Up
SGPP2	1.772672967	2.540438167	7.670444393	0.000828	0.02143874	-0.34461652	Up
C10ORF65	2.534759605	3.178776068	7.030097182	0.001208	0.026890131	-0.78543207	Up
TMEFF2	1.642000188	3.677401042	6.901837952	0.001308	0.027930805	-0.87796903	Up
UPK1B	1.451696202	2.8422269	6.736774554	0.001451	0.029608357	-0.99927656	Up
S100A4	3.447683894	5.957251779	6.425016075	0.001775	0.033314821	-1.23548384	Up
AOAH	1.907506477	3.653583998	6.338972788	0.00188	0.03438737	-1.30237174	Up
FOLR1	1.835509449	3.808853432	5.222184833	0.004208	0.056368147	-2.24440307	Up
KIAA1727	1.622395213	4.480804798	5.10688588	0.004607	0.059328254	-2.3501893	Up
RARRES3	1.499904126	5.143872218	4.814542355	0.005837	0.068732904	-2.62629155	Up
DTX4	1.297304199	4.966580474	4.185599416	0.010078	0.096101735	-3.26091734	Up
MFGE8	1.22865476	7.107373458	3.962409701	0.012394	0.109181962	-3.50019902	Up
CLDN2	1.832969106	4.656448303	3.603110124	0.017564	0.136071363	-3.90147006	Up
GOLSYN	1.419600758	4.173530047	3.048496874	0.031303	0.197422949	-4.55946496	Up
