# Supplementary material for: VHL-HIF-2α axis-induced SEMA6A upregulation stabilized β-catenin to drive clear cell renal cell carcinoma progression
Source: Cell Death Dis. 2023 Feb 4;14(2):83. doi: 10.1038/s41419-023-05588-4 (PMC9899268; doi:10.1038/s41419-023-05588-4)
Supplement: Supplementary file 11 — Supplementary Table4 [file 41419_2023_5588_MOESM11_ESM.pdf]

Table S4.List of genes that were down-regulated in the HA-VHL reintroduced group compared with the control group.

Note:GSE32297, logFC<-1.2,P<0.05

Gene	logFC	AveExpr	t	P.Value	adj.P.Val	B	threshold
MME	-3.55031	3.939259	-113.022	3.57E-09	2.69E-05	9.171126	Down
PSCDBP	-6.41584	6.063564	-75.0809	2.39E-08	0.000104	8.710213	Down
EGLN3	-4.06512	5.089417	-69.3689	3.45E-08	0.00012	8.582701	Down
MGC23985	-5.64419	4.633919	-56.4161	9.00E-08	0.00026	8.177462	Down
IRS2	-1.83932	11.43558	-54.6689	1.04E-07	0.00026	8.10601	Down
PLAC8	-1.5111	9.688436	-46.9859	2.10E-07	0.000408	7.724	Down
MTUS1	-2.46753	4.419019	-44.3	2.76E-07	0.000455	7.558273	Down
CXCR7	-4.27506	8.622418	-42.0575	3.52E-07	0.000478	7.40398	Down
MACROD2	-1.2599	8.115436	-35.9613	7.27E-07	0.000634	6.893997	Down
VEGFA	-1.28913	9.737402	-34.1939	9.18E-07	0.000746	6.715961	Down
LCP1	-3.82	5.726523	-33.9581	9.48E-07	0.000746	6.691005	Down
C1QL1	-4.18324	10.13475	-33.0846	1.07E-06	0.000747	6.595929	Down
ADM	-1.93332	10.20611	-30.2664	1.62E-06	0.001007	6.258484	Down
F3	-2.86787	11.00857	-25.6535	3.47E-06	0.001427	5.583742	Down
MYEOV	-1.52381	9.633754	-24.9437	3.95E-06	0.001427	5.463594	Down
SCG5	-1.78017	10.37944	-23.4343	5.27E-06	0.001586	5.190986	Down
ZCCHC5	-2.69757	3.767123	-22.6341	6.19E-06	0.001682	5.036235	Down
ARTN	-2.1657	5.976594	-22.6285	6.19E-06	0.001682	5.035131	Down
NGFB	-2.08077	4.059431	-22.0567	6.97E-06	0.001728	4.919803	Down
MALL	-1.65702	9.317231	-22.0083	7.04E-06	0.001728	4.909848	Down
FAM83F	-1.80658	2.977647	-20.3844	1.00E-05	0.002107	4.558006	Down
APOL1	-1.25152	11.82319	-19.8843	1.12E-05	0.002216	4.442126	Down
ITGB8	-1.39259	6.197818	-17.7374	1.90E-05	0.003041	3.898637	Down
ENO2	-1.2566	9.563856	-15.9756	3.07E-05	0.003882	3.388075	Down
GAL3ST1	-1.51108	9.832692	-15.6871	3.34E-05	0.003953	3.298067	Down
SLAMF8	-2.67614	6.233912	-14.4689	4.83E-05	0.004669	2.895507	Down
NCF2	-2.81245	5.854818	-13.9028	5.79E-05	0.005153	2.694989	Down
HSF2BP	-2.64487	5.889301	-13.5395	6.53E-05	0.005531	2.561437	Down
HNRPLL	-1.50822	6.573482	-13.4108	6.82E-05	0.00566	2.513129	Down
ODZ2	-1.86381	9.296533	-12.5145	9.34E-05	0.006764	2.162116	Down
KIAA1909	-1.58264	5.987052	-12.4448	9.58E-05	0.006822	2.133664	Down
HBEGF	-1.8256	8.966851	-12.1475	0.0001068	0.007143	2.010402	Down
SPOCD1	-1.48219	8.597294	-12.0934	0.000109	0.007206	1.987625	Down
SEMA6A	-2.29195	6.795616	-12.0596	0.0001104	0.007243	1.973349	Down
ZNF185	-2.49153	6.623684	-11.2012	0.0001541	0.008578	1.595555	Down
AYTL1	-1.35411	8.296217	-10.7164	0.0001881	0.009297	1.368583	Down
GPR176	-1.23437	8.15986	-10.2551	0.0002291	0.010331	1.142593	Down
WNT5A	-1.40286	7.474213	-9.48711	0.0003244	0.012396	0.742624	Down
LTBP1	-2.0709	3.785142	-9.37104	0.0003427	0.012725	0.679398	Down
SLITRK2	-1.97375	3.371938	-7.51908	0.0009031	0.022642	-0.44576	Down
LRIG1	-1.46868	4.475934	-7.28256	0.0010373	0.024563	-0.60753	Down
EVI2A	-1.49508	4.955744	-6.82075	0.0013757	0.028738	-0.93724	Down
FGF5	-1.48143	4.679267	-6.66696	0.0015167	0.030354	-1.05136	Down
ELL2	-1.6227	8.909846	-6.51368	0.0016749	0.032156	-1.16734	Down
BAI2	-2.16958	5.465097	-6.33916	0.0018796	0.034387	-1.30223	Down
PLXNA2	-2.20299	3.701772	-5.61144	0.0031325	0.047125	-1.89962	Down

HCG4	-1.42403	7.145761	-5.08553	0.0046853	0.05988	-2.36997	Down
FABP6	-1.38837	6.080911	-3.56956	0.0181635	0.139267	-3.93995	Down
C1ORF51	-1.211	3.446964	-3.38366	0.0219469	0.157094	-4.15634	Down
