# Supplementary material for: VHL-HIF-2α axis-induced SEMA6A upregulation stabilized β-catenin to drive clear cell renal cell carcinoma progression
Source: Cell Death Dis. 2023 Feb 4;14(2):83. doi: 10.1038/s41419-023-05588-4 (PMC9899268; doi:10.1038/s41419-023-05588-4)
Supplement: Supplementary file 12 — Supplementary Table5 [file 41419_2023_5588_MOESM12_ESM.pdf]

Table S5.The correlation between SEMA6A and 99 hypoxia metagenes in the TCGA-KIRC database.

Note: The 99 genes are from the research by Adrian L. Harris and Francesca M. Buffa. DOI: 10.1158/0008-5472.CAN-06-3322

Gene	correlation	P-value
NDRG1	0.482	2.20E-16
PGAM1	0.472	2.20E-16
BMS1L	0.47	0
BNIP3	0.445	2.20E-16
MGC14560	0.42	9.5e-14
LDHA	0.402	2.20E-16
CORO1C	0.401	2.20E-16
VEGF	0.38	0
LRP2BP	0.378	2.20E-16
RNPS1	0.365	2.20E-16
VEZT	0.349	2.20E-16
XPO5	0.344	3.40E-16
P4HA1	0.34	7.90E-16
GMFB	0.339	9.65E-16
PGK1	0.334	2.42E-15
PPP4R1	0.334	2.90E-15
EIF2S1	0.334	2.53E-15
C20orf20	0.32	3.6e-08
C15orf25	0.32	2.6e-08
VAPB	0.316	8.31E-14
AK3	0.304	7.46E-13
KIAA1393	0.3	2.50E-12
PLEKHG3	0.297	3.00E-12
ALDOA	0.296	3.39E-12
PSMB7	0.289	1.14E-11
TPI1	0.283	3.23E-11
BCAR1	0.28	5.34E-11
IMP-2	0.28	1.5e-06
KCTD11	0.278	7.53E-11
PAWR	0.273	1.66E-10
PSMD2	0.269	2.98E-10
SLC6A8	0.26	1.19E-09
NUDT15	0.254	2.95E-09
PPARD	0.248	6.59E-09
SLC16A1	0.233	5.55E-08
SMILE	0.23	6.6e-05
MRPS17	0.228	1.07E-07
GSS	0.225	1.51E-07
PFKFB4	0.209	1.26E-06
DPM2	0.209	1.16E-06
ANGPTL4	0.207	1.51E-06
DKFZP564D166	0.19	0.0013
MRPL14	0.189	1.22E-05
HES2	0.187	1.47E-05
TIMM23	0.183	2.28E-05
SNX24	0.181	2.85E-05
CA12	0.179	3.46E-05

RAN	0.176	4.38E-05
TPBG	0.172	6.64E-05
TPD52L2	0.172	6.64E-05
SLC2A1	0.17	8.54E-05
MNAT1	0.165	0.000133
RNF24	0.159	0.000243
CA9	0.159	0.000245
PYGL	0.1555	0.000336
SIP1	0.13	0.027
S100A10	0.126	0.00867
TUBB2	0.12	0.039
ANLN	0.118	0.00638
RUVBL2	0.112	0.0101
MGC2408	0.11	0.064
SLC6A10	0.087	0.046
MTX1	0.0801	0.0652
CDCA4	0.0771	0.0758
PVR	0.0677	0.119
MGC17624	0.059	0.32
ANKRD9	0.0376	0.387
PSMA7	0.0368	0.398
SLCO1B3	0.0354	0.415
B4GALT2	0.0311	0.475
SPTB	0.0167	0.701
HOMER1	0.0122	0.779
AD-003	0.009	0.88
ADORA2B	0.00809	0.853
LDLR	-0.0321	0.461
PLAU	-0.038	0.382
IL8	-0.041	0.49
COL4A5	-0.0433	0.319
GAPD	-0.046	0.3
TEAD4	-0.0477	0.272
PGF	-0.0584	0.179
PDZK11	-0.062	0.16
TMEM30B	-0.0913	0.0355
HSPC163	-0.095	0.029
MIF	-0.118	0.00656
Kua	-0.12	0.0049
CTEN	-0.14	0.0016
PPP2CZ	-0.17	1.00E-04
S100A3	-0.175	5.08E-05
C14orf156	-0.18	0.0022
NME1	-0.2	3.44E-06
PTGFRN	-0.212	8.01E-07
KRT17	-0.228	1.04E-07
TFAP2C	-0.611	2.14E-13
AFARP1	NA	NA
MGC2654	NA	NA
HIG2	NA	NA
LOC149464	NA	NA
LOC56901	NA	NA
