# Supplementary material for: VHL-HIF-2α axis-induced SEMA6A upregulation stabilized β-catenin to drive clear cell renal cell carcinoma progression
Source: Cell Death Dis. 2023 Feb 4;14(2):83. doi: 10.1038/s41419-023-05588-4 (PMC9899268; doi:10.1038/s41419-023-05588-4)
Supplement: Supplementary file 13 — Supplementary Table6 [file 41419_2023_5588_MOESM13_ESM.pdf]

Table S6.The correlation between SEMA6A and 26 hypoxia metagenes in the TCGA-KIRC database.

Note: The 26 genes are from the research by Adrian L. Harris and Francesca M. Buffa. DOI: 10.1158/1078-0432.CCR-13-0542

Gene	correlation	P-value
PGAM1	0.41	0
VEGFA	0.38	0
LDHA	0.37	0
ENO1	0.32	1e-13
PGK1	0.31	4.4e-13
P4HA1	0.26	1.2e-09
ALDOA	0.23	1.8e-07
KCTD11	0.22	3.9e-07
TPI1	0.22	3.2e-07
SLC2A1	0.16	0.00019
GNAI1	0.14	0.00089
ANGPTL4	0.12	0.0043
CA9	0.12	0.0076
DCBLD1	0.088	0.044
SLC16A1	0.039	0.38
SDC1	−0.086	0.05
COL4A6	−0.088	0.044
KRT17	-0.089	0.043
ANLN	-0.099	0.023
FOSL1	-0.13	0.0036
BNC1	−0.15	0.00038
CDKN3	−0.16	0.00027
FAM83B	−0.16	0.00023
C20orf20	NA	NA
HIG2	NA	NA
MPRS17	NA	NA
