# Supplementary material for: VHL-HIF-2α axis-induced SEMA6A upregulation stabilized β-catenin to drive clear cell renal cell carcinoma progression
Source: Cell Death Dis. 2023 Feb 4;14(2):83. doi: 10.1038/s41419-023-05588-4 (PMC9899268; doi:10.1038/s41419-023-05588-4)
Supplement: Supplementary file 14 — Supplementary Table7 [file 41419_2023_5588_MOESM14_ESM.pdf]

Table S7.List of genes that were up-regulated in the Sema6A_KO group compared with the control group.

NOTE:GSE79683, logFC>1.2,P<0.05

Gene	logFC	AveExpr	t	P.Value	adj.P.Val	B	threshold
Lyz2	3.011988	7.06184	21.33497	1.32E-06	0.010019	5.101849	Up
Tyrobp	2.479507	8.918806	18.2532	3.15E-06	0.010019	4.64194	Up
A730054J2	3.510431	7.560482	17.90233	3.51E-06	0.010019	4.579436	Up
Cebpa	1.662688	7.369944	17.63773	3.82E-06	0.010019	4.530694	Up
Ly86	2.442382	8.794371	17.62522	3.83E-06	0.010019	4.528356	Up
Kif26b	2.1913	5.795591	17.60855	3.85E-06	0.010019	4.525233	Up
Nrgn	1.887311	6.18947	17.38453	4.13E-06	0.010019	4.48271	Up
Gpr39	2.997403	7.869948	16.58434	5.37E-06	0.011707	4.321893	Up
Fcgr3	2.094164	7.452621	15.82764	6.95E-06	0.013453	4.155879	Up
P2ry6	1.872495	6.22404	15.64778	7.40E-06	0.013453	4.114262	Up
AW551984	1.507488	11.09694	15.14273	8.87E-06	0.013821	3.992653	Up
Bcl2a1a /	1.888828	7.168658	13.66531	1.56E-05	0.02109	3.592291	Up
Pld4	1.468813	6.630544	13.5986	1.60E-05	0.02109	3.572482	Up
Lypd1	2.793583	9.05897	13.53679	1.64E-05	0.02109	3.553979	Up
Pcdh17	2.264133	9.641055	13.1662	1.91E-05	0.023193	3.440073	Up
Evi2a	2.183877	6.148901	12.4999	2.54E-05	0.026936	3.221653	Up
Tmcc3	1.315753	10.13685	6.932619	0.000585	0.07662	0.396384	Up
Clec7a	1.908224	3.89307	6.840652	0.000626	0.078654	0.328338	Up
Klhl14	1.425818	9.243447	6.832236	0.00063	0.078654	0.322064	Up
Pdyn	1.731275	6.237481	6.790206	0.00065	0.079338	0.29061	Up
P2ry13	1.363306	6.58848	6.745108	0.000673	0.080118	0.256638	Up
Mpeg1	2.470918	8.256772	6.736118	0.000677	0.080118	0.249837	Up
A930009L(	1.402744	6.002158	6.73131	0.00068	0.080118	0.246197	Up
Sphkap	1.639313	11.11555	6.674452	0.00071	0.081494	0.202943	Up
Ppp1r17	2.033706	5.200936	6.517567	0.000801	0.084204	0.081636	Up
Hpse	1.362552	5.801666	6.488409	0.000819	0.084204	0.058769	Up
Cd180	1.705659	6.178929	6.482723	0.000823	0.084204	0.054298	Up
Gm9947	1.373401	5.765751	6.476867	0.000827	0.084204	0.049689	Up
Pde1c	2.362529	10.03704	6.337042	0.000923	0.087114	-0.06159	Up
Slc44a2	1.39933	9.580962	6.33285	0.000926	0.087114	-0.06496	Up
Rab32	1.487332	6.174388	6.286217	0.000961	0.087324	-0.10263	Up
Flrt1	1.621046	8.723532	6.208834	0.001022	0.090772	-0.16574	Up
Crhbp	1.474185	4.626526	6.139723	0.001081	0.091487	-0.22274	Up
Ccl3	1.73096	4.76666	6.133969	0.001086	0.091487	-0.22751	Up
Ccr5	1.4995	4.668102	6.119999	0.001099	0.092178	-0.23912	Up
Amz1	1.392136	6.029115	5.962805	0.00125	0.099803	-0.37144	Up
Gpr34	1.703617	6.393113	5.959863	0.001254	0.099803	-0.37395	Up
Khdrbs3	1.907709	7.15302	5.947855	0.001266	0.09993	-0.3842	Up
Klhl1	2.47359	5.278744	5.900287	0.001318	0.101488	-0.42497	Up
Ctss	1.885417	10.14889	5.895616	0.001323	0.101488	-0.42899	Up
Fzd5	1.322216	6.029574	5.880861	0.001339	0.101541	-0.44171	Up
Ms4a6d	2.310949	5.049907	5.830443	0.001397	0.103686	-0.48539	Up
Krt19	2.384234	5.992494	5.818438	0.001412	0.104389	-0.49584	Up
9830166K(	1.374529	3.830444	5.757455	0.001487	0.105232	-0.54922	Up
Dclk3	1.712139	6.901704	5.672936	0.001599	0.107904	-0.62405	Up
Ptprc	1.607295	5.142083	5.658266	0.001619	0.107904	-0.63714	Up
LOC102631	1.537383	5.336716	5.615056	0.001681	0.10814	-0.67587	Up

Naip5	1.689084	4.040137	5.587334	0.001722	0.10814	-0.70085	Up
Pycard	1.325964	7.028502	5.547687	0.001783	0.10814	-0.73676	Up
Csf2rb	1.665808	5.59898	5.482543	0.001889	0.110076	-0.79626	Up
Rassf4	1.491523	6.630989	5.38005	0.002069	0.113998	-0.89109	Up
Rab3b	1.659524	9.910076	5.37438	0.00208	0.114291	-0.89638	Up
1700016K1	2.031385	6.87142	5.36128	0.002105	0.114627	-0.90862	Up
Kirrel	1.503939	5.726378	5.359977	0.002107	0.114627	-0.90984	Up
Kcnk6	1.642737	3.981477	5.334809	0.002155	0.11553	-0.93343	Up
Grm1	1.468584	9.020959	5.179173	0.002485	0.125175	-1.08138	Up
Spats2l	1.315357	8.615188	5.165801	0.002515	0.125571	-1.09426	Up
Ntsr2	1.461059	6.117837	5.155692	0.002539	0.125887	-1.10402	Up
Spock1	1.306634	9.167192	5.070593	0.002748	0.130626	-1.18674	Up
Igk-V28 /	1.947813	5.17859	5.062085	0.00277	0.131006	-1.19507	Up
P2ry12	1.394266	5.577866	4.810532	0.003522	0.146447	-1.44639	Up
Maob	1.664368	5.983826	4.761823	0.003693	0.147023	-1.49619	Up
Apoc1	1.613605	6.290473	4.688176	0.00397	0.150571	-1.57219	Up
Tnfrsf1b	1.371439	6.574282	4.668321	0.004049	0.151342	-1.59283	Up
Baiap3	1.623621	7.024495	4.667593	0.004052	0.151342	-1.59359	Up
Gm3515 //	1.676169	4.423402	4.664949	0.004062	0.151479	-1.59634	Up
Themis	1.879265	4.483515	4.661391	0.004076	0.151755	-1.60005	Up
Slc35f4	1.354146	6.584309	4.59778	0.004343	0.156345	-1.66666	Up
Rspo1	2.379857	5.105976	4.510968	0.004739	0.16281	-1.75861	Up
A330068G1	1.515261	7.510695	4.460966	0.004986	0.167337	-1.81213	Up
Kirrel3	1.700599	10.17531	4.336049	0.005669	0.177633	-1.9476	Up
Unc5d	2.180901	7.074928	4.314202	0.005799	0.179193	-1.97155	Up
Fam107a	2.563343	7.035026	4.294266	0.005921	0.180387	-1.99348	Up
Bcl11b	1.606324	8.232833	4.182184	0.006661	0.187748	-2.11796	Up
Sox1	1.392625	8.793855	4.151792	0.00688	0.189696	-2.15207	Up
Adssl1	1.61663	6.654576	4.142961	0.006945	0.190373	-2.16201	Up
Itgb2	1.426135	4.911332	4.10354	0.007244	0.19392	-2.20653	Up
Grp	1.819116	5.643372	4.074081	0.007476	0.197047	-2.23997	Up
Hcrtr1	1.306541	5.536054	4.070652	0.007504	0.197047	-2.24388	Up
Htr2c	1.705165	6.945922	4.056756	0.007617	0.19849	-2.25971	Up
Glra3	1.398387	7.738347	4.043947	0.007723	0.199149	-2.27433	Up
Fam46a	1.782052	5.680531	3.907929	0.008958	0.213172	-2.43126	Up
Kcnab1	1.630863	7.510518	3.855597	0.009491	0.217301	-2.49244	Up
Pcsk2	1.815723	10.57699	3.853175	0.009517	0.217394	-2.49528	Up
Syndig1	1.453181	7.652725	3.831828	0.009745	0.220076	-2.52038	Up
Pmch	2.694095	8.081547	3.803005	0.010064	0.223721	-2.55438	Up
Synpr	1.445614	7.889825	3.796526	0.010137	0.223817	-2.56204	Up
Fam183b	1.404465	5.842123	3.718628	0.011064	0.233357	-2.6547	Up
Tlr1	2.056998	4.993363	3.71385	0.011124	0.233791	-2.66041	Up
Eif2ak2	1.437121	7.312054	3.710831	0.011162	0.233914	-2.66402	Up
LOC10264(	1.623679	4.101222	3.674292	0.011635	0.239284	-2.70787	Up
Mbp	1.561779	9.663763	3.642955	0.012058	0.24176	-2.74564	Up
Fzd7	1.548689	6.161452	3.614836	0.012452	0.246277	-2.77966	Up
Esr1	1.324844	4.586037	3.510886	0.014039	0.257583	-2.90654	Up
Sema3c	2.059004	6.87368	3.393102	0.016117	0.270386	-3.05232	Up
Fyb	1.852586	6.64712	3.388566	0.016203	0.270386	-3.05798	Up
Eno4	2.227788	5.795072	3.342384	0.017115	0.274775	-3.11575	Up
Trhr	1.328521	3.955419	3.30247	0.017949	0.277887	-3.16593	Up
Cidea	1.410584	6.225956	3.296072	0.018087	0.277887	-3.174	Up
Gpr83	1.806029	5.495082	3.226301	0.019668	0.286418	-3.26234	Up
Psmb8	1.38538	6.694264	3.172233	0.020999	0.292071	-3.33127	Up

Timd2	1.905109	4.863622	3.163727	0.021217	0.293683	-3.34215	Up
Pdzrn3	1.504444	8.821921	3.161294	0.02128	0.293891	-3.34526	Up
Chst11 //	1.580598	7.35299	3.137849	0.021896	0.296872	-3.37531	Up
Trim30a	2.019359	6.56821	3.130712	0.022088	0.297812	-3.38448	Up
Ramp3	1.70889	7.384162	3.100813	0.022911	0.30383	-3.42294	Up
Pifo	1.585263	3.950284	3.085406	0.023348	0.304337	-3.4428	Up
Clca1 ///	1.578064	5.670047	3.066487	0.023898	0.307088	-3.46724	Up
Padi2	1.465164	6.855485	2.988331	0.026323	0.319433	-3.56866	Up
Abca9	1.353824	6.469052	2.979927	0.026599	0.320493	-3.57962	Up
Serpinb11	1.507034	3.980345	2.94512	0.027779	0.32671	-3.62506	Up
Aldh1a7	2.309752	5.161449	2.921659	0.028606	0.330702	-3.65578	Up
Dcstamp	1.606118	3.7291	2.915455	0.028829	0.331336	-3.66391	Up
Npy	2.454268	7.9812	2.778089	0.03429	0.354524	-3.84504	Up
C5ar1	1.396647	6.294858	2.765	0.034867	0.354949	-3.8624	Up
Ccl12	1.557034	5.574525	2.707588	0.037522	0.362689	-3.93873	Up
Sst	2.079171	7.679634	2.644696	0.040683	0.371834	-4.02268	Up
Clybl	1.388572	8.853624	2.608358	0.042639	0.379819	-4.07132	Up
Chodl	1.352107	5.609502	2.551419	0.045911	0.392182	-4.14771	Up
Calb1	1.393085	9.81306	2.538763	0.046674	0.394761	-4.16471	Up
Phf11d	1.500336	3.629541	2.528648	0.047294	0.396811	-4.17831	Up
Doc2b	2.002353	6.235828	2.514739	0.04816	0.39789	-4.19702	Up
Nlrc5	1.577061	6.445934	2.50795	0.048589	0.398786	-4.20616	Up
Cacng3	1.496531	7.621438	2.498319	0.049205	0.40067	-4.21912	Up
