# Supplementary material for: VHL-HIF-2α axis-induced SEMA6A upregulation stabilized β-catenin to drive clear cell renal cell carcinoma progression
Source: Cell Death Dis. 2023 Feb 4;14(2):83. doi: 10.1038/s41419-023-05588-4 (PMC9899268; doi:10.1038/s41419-023-05588-4)
Supplement: Supplementary file 15 — Supplementary Table8 [file 41419_2023_5588_MOESM15_ESM.pdf]

Table S8.List of genes that were down-regulated in the Sema6A_KO group compared with the control group.

NOTE:GSE79683, logFC<-1.2,P<0.05

Gene	logFC	AveExpr	t	P.Value	adj.P.Val	B	threshold
Sema6a	-2.66466	9.430814	-21.986	1.12E-06	0.010019	5.181823	Down
Cck	-2.10256	11.02857	-20.7986	1.53E-06	0.010019	5.031939	Down
Gsg1l	-2.48627	10.3519	-15.3198	8.32E-06	0.013821	4.036105	Down
Muc15	-1.63922	10.08774	-12.376	2.68E-05	0.026936	3.178979	Down
Snx31	-2.34632	5.987953	-11.191	4.64E-05	0.030688	2.734996	Down
Kcnn1	-1.58112	9.478733	-11.0032	5.09E-05	0.031457	2.658143	Down
Pcdha1 //	-1.4791	9.469391	-10.9626	5.19E-05	0.031457	2.641267	Down
Dkk4	-1.98937	8.270216	-10.1148	8.01E-05	0.037199	2.267566	Down
Ism1	-2.94357	5.993137	-10.021	8.43E-05	0.037513	2.223542	Down
BC100530	-3.16465	6.409688	-9.93678	8.82E-05	0.038468	2.183529	Down
S100a8	-2.45159	5.90271	-9.64871	0.000103	0.039508	2.043157	Down
Egflam	-1.52662	6.888752	-9.30645	0.000125	0.042172	1.868991	Down
Ptpn22	-2.39105	6.957045	-9.21373	0.000132	0.043001	1.820369	Down
Kcna1	-1.70954	8.4301	-9.17494	0.000135	0.043332	1.799838	Down
Stfa3	-1.70038	4.54382	-8.87068	0.000162	0.048959	1.634888	Down
Lgr5	-3.00266	5.114194	-8.80429	0.000168	0.049572	1.597945	Down
Tex14	-1.43463	7.200862	-8.06433	0.000267	0.058274	1.161665	Down
S100a9	-2.31592	7.244113	-8.00806	0.000277	0.058878	1.126549	Down
Itga9	-1.68141	6.366052	-7.59813	0.000365	0.067988	0.861831	Down
Osbpl3	-1.32651	8.418171	-7.55613	0.000375	0.067988	0.833793	Down
Smyd1	-1.44984	9.308678	-7.13215	0.000506	0.071626	0.540816	Down
Cnr2	-1.63402	6.085311	-6.87857	0.000609	0.078052	0.35651	Down
Hdc	-1.91238	8.953525	-5.83235	0.001395	0.103686	-0.48373	Down
Dlk1	-1.84277	9.645117	-5.71256	0.001545	0.106573	-0.58885	Down
9330101J(	-1.32525	4.098265	-5.68707	0.001579	0.107904	-0.61147	Down
Tmprss3	-1.40793	5.5231	-5.54736	0.001784	0.10814	-0.73706	Down
Sorcs3	-1.45885	9.585044	-5.29037	0.002244	0.116279	-0.97531	Down
Stfa2l1	-2.0295	5.755156	-5.27053	0.002285	0.117559	-0.9941	Down
Cdh1	-1.45865	4.651853	-5.10681	0.002657	0.128395	-1.1514	Down
A730009L(	-1.6161	4.785916	-5.06815	0.002755	0.130641	-1.18913	Down
Fabp4	-2.10474	5.994916	-5.01487	0.002896	0.133577	-1.2415	Down
2900011F(	-1.37109	4.18797	-4.98308	0.002985	0.135929	-1.27295	Down
Stxbp6	-1.515	11.04273	-4.93837	0.003114	0.139216	-1.31745	Down
Scel	-1.40849	4.784848	-4.86786	0.003332	0.143083	-1.38825	Down
Adcy2	-1.43315	10.3217	-4.81994	0.00349	0.146239	-1.43681	Down
Cdca7	-1.55507	9.158531	-4.76452	0.003683	0.147023	-1.49342	Down
Snhg18	-1.39357	7.900269	-4.75096	0.003732	0.147235	-1.50734	Down
Ptgfr	-1.31187	4.1511	-4.70206	0.003916	0.150133	-1.5578	Down
Ndnf	-2.17838	5.280903	-4.69469	0.003944	0.150483	-1.56543	Down
4930406D1	-1.34719	6.735946	-4.61196	0.004282	0.156228	-1.65176	Down
Ibsp	-2.27651	5.849706	-4.06666	0.007536	0.197362	-2.24842	Down
Wif1	-1.5094	5.588455	-3.98942	0.008194	0.204225	-2.33688	Down
Bmp6	-1.39459	8.055626	-3.82909	0.009775	0.220291	-2.5236	Down
Fam83f	-1.42102	7.664412	-3.66262	0.01179	0.239593	-2.72192	Down
Fam19a4	-1.38048	9.76958	-3.46558	0.014801	0.261005	-2.96236	Down
Shb	-1.31668	9.414164	-3.46494	0.014812	0.261005	-2.96316	Down
Col1a1	-1.94328	9.015252	-3.3891	0.016193	0.270386	-3.05731	Down

Nexn	-1.34193	8.193076	-3.2673	0.018721	0.281458	-3.21033	Down
Dcdc2a	-1.561	7.1994	-3.08062	0.023486	0.304603	-3.44898	Down
Ptgds	-1.71597	6.93148	-2.58455	0.043976	0.384654	-4.10323	Down
