# Supplementary material for: VHL-HIF-2α axis-induced SEMA6A upregulation stabilized β-catenin to drive clear cell renal cell carcinoma progression
Source: Cell Death Dis. 2023 Feb 4;14(2):83. doi: 10.1038/s41419-023-05588-4 (PMC9899268; doi:10.1038/s41419-023-05588-4)
Supplement: Supplementary file 16 — Original Data File [file 41419_2023_5588_MOESM16_ESM.pptx]

## Slide 1
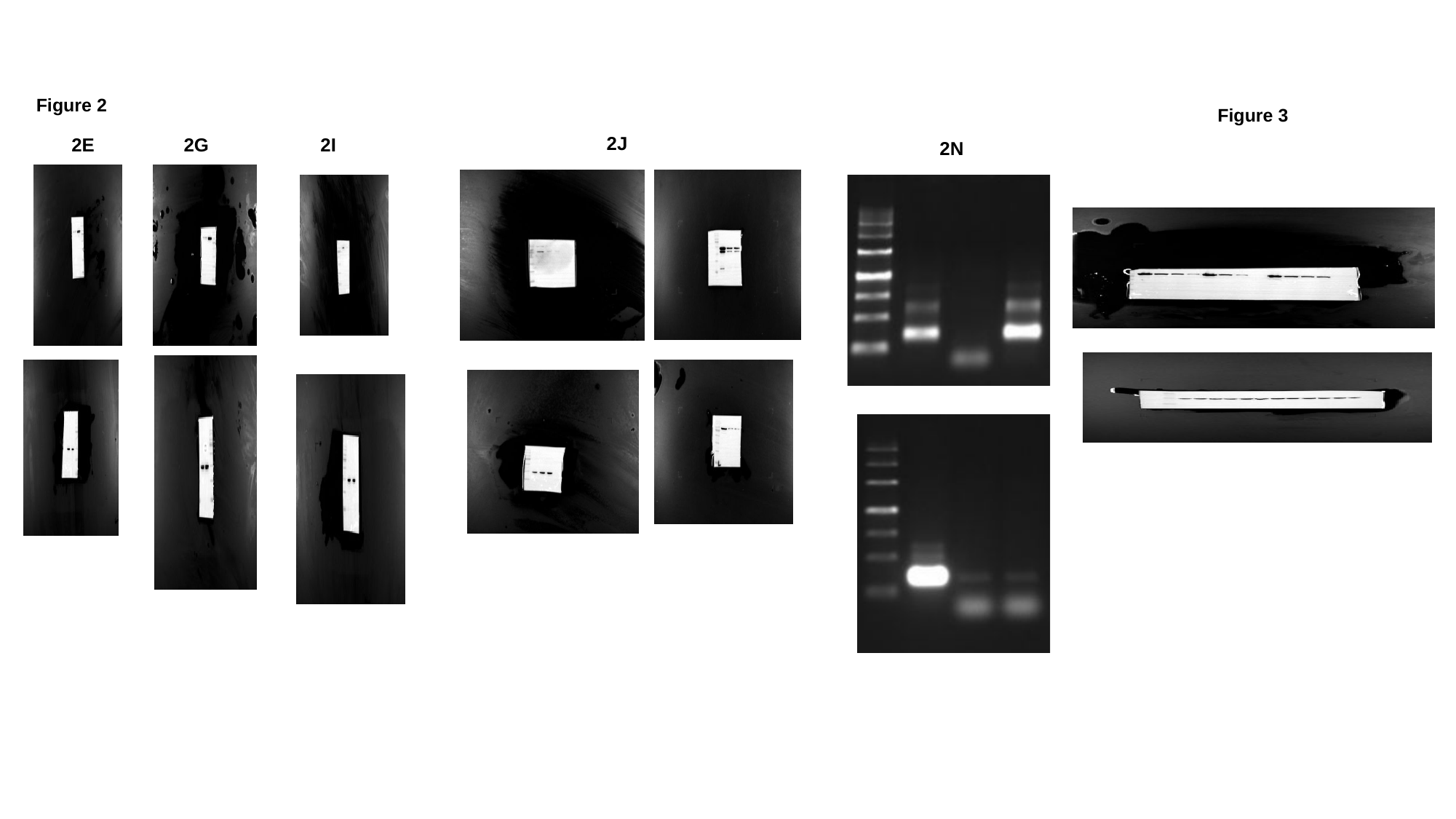

Figure 2
Figure 3
2J
2E 2G
2I
2N

## Slide 2
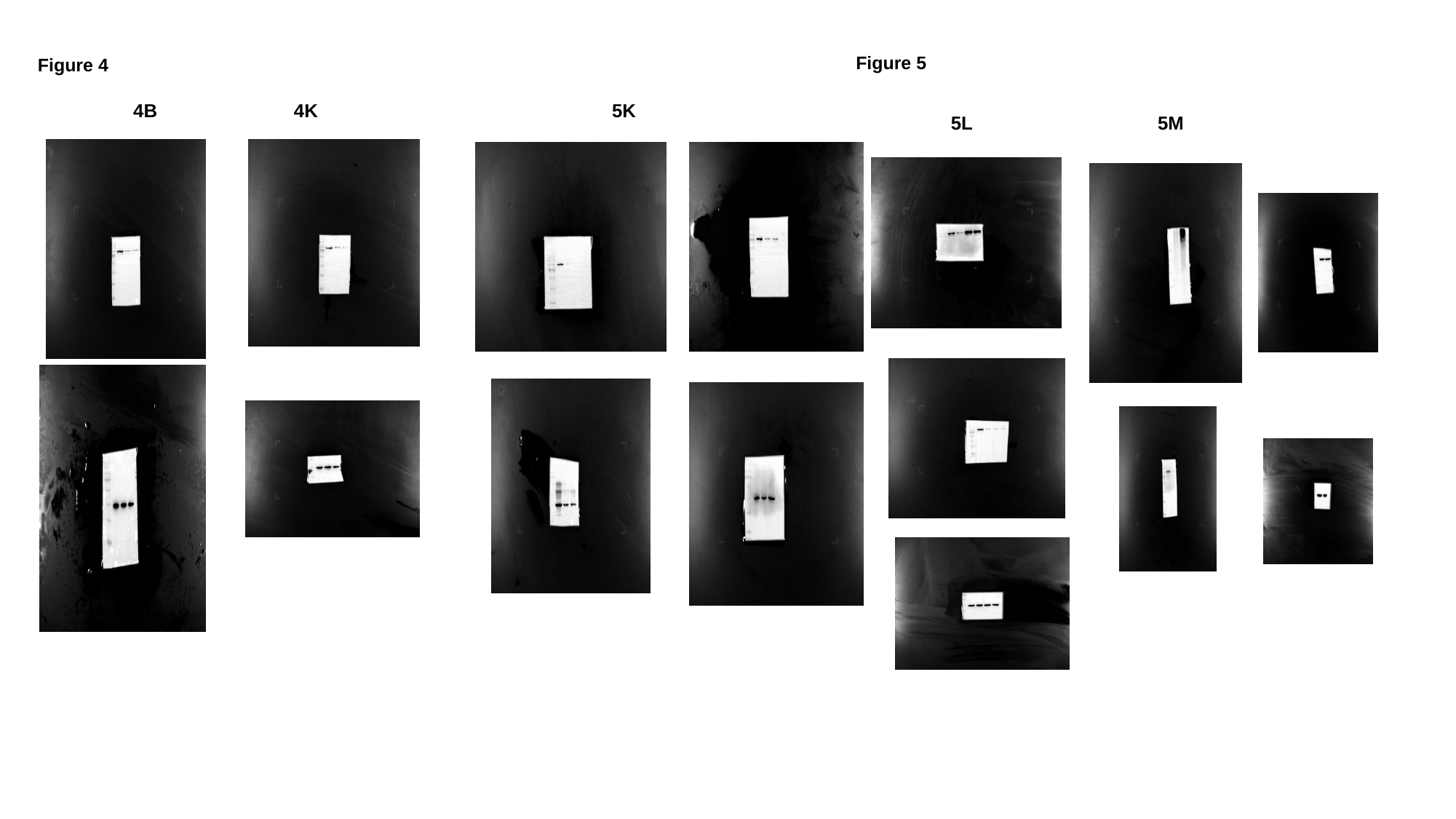

Figure 5
Figure 4
4B 4K
5K
5L
5M

## Slide 3
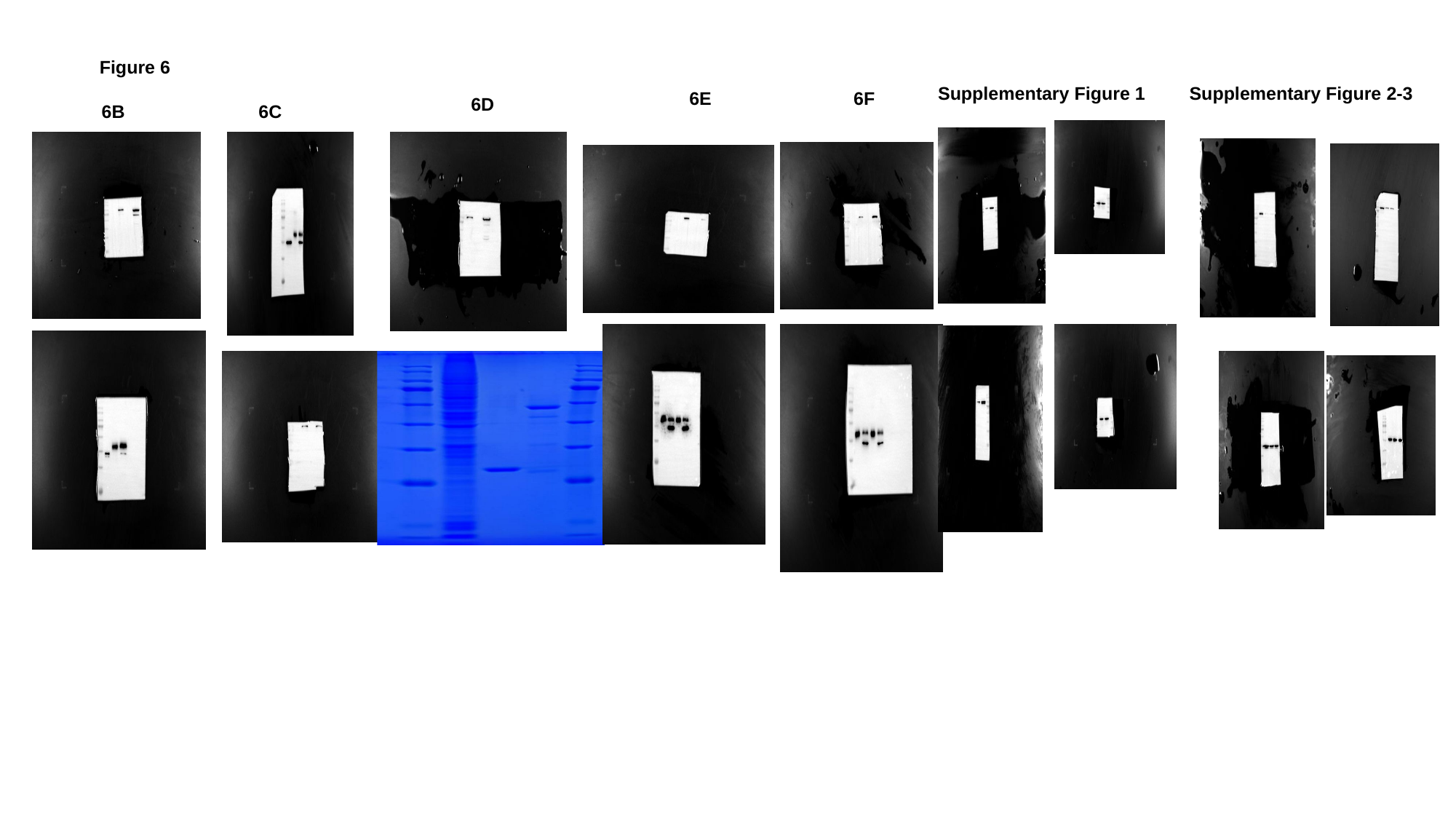

Figure 6
Supplementary Figure 1
Supplementary Figure 2-3
6F
6E
6D
6C
6B
